# Supplementary material for: Predictive modeling for trustworthiness and other subjective text properties in online nutrition and health communication
Source: PLoS One. 2020 Aug 6;15(8):e0237144. doi: 10.1371/journal.pone.0237144 (PMC7410325; doi:10.1371/journal.pone.0237144)
Supplement: S1 File — (DOCX) [file pone.0237144.s001.docx]

**Supporting Information** for a manuscript “Kauttonen Janne et al.: *Predictive modeling for trustworthiness and other subjective text properties in online nutrition and health communication*”

**Content:**

[1. Pipeline design and feature extraction methods 2](#_Toc37354326)

[2. Regression algorithms and hyperparameters 3](#_Toc37354327)

[3. Ensemble learning models and pooling of fit coefficients 4](#_Toc37354328)

[4. Exploratory analysis of linear fit coefficients 5](#_Toc37354329)

[5. Text rating estimation and method comparison 8](#_Toc37354330)

[6. Histograms of behavioral variables 10](#_Toc37354331)

[7. Parameters and features of the top models 10](#_Toc37354332)

[8. Analysis of word2vec document embeddings 12](#_Toc37354333)

[9. Illustrations of the linear prediction process 15](#_Toc37354334)

[References 19](#_Toc37354335)

# 1. Pipeline design and feature extraction methods

Here we provide a detailed summary, including short history and motivation, of our pipeline and the three feature extraction methods we applied. The design of our pipeline was inspired by such tools as EASE (<https://github.com/edx/ease>) and LightSIDE ([http://ankara.lti.cs.cmu.edu/side](http://ankara.lti.cs.cmu.edu/side/)) and similar systems [1]. We represented texts using a combination of *n*-grams, word2vec document embeddings and a set of handcrafted features. Combining feature types has been successful in various recent NLP studies [2–11]. Our three feature extraction methods included:

**1. Bag-of-*n*-grams:** Using *n*-grams, a text is expressed as a combination of nearest-neighbor tokens (e.g., words or punctuations), such that individual tokens are 1-grams (unigrams), token pairs are 2-grams (bigrams) and so forth. Each unique *n*-gram is represented as a vector using one-hot-encoding. As a result, a text can be converted into an integer vector, a *bag-of-n-grams*, where each element represents the count of a specific *n*-gram. In order to compensate for document length and relative frequency of various n-gram, these vectors are often scaled with *term-frequencies* (TF) and *inverse document frequencies* (IDF), which together form TF-IDF scaling scheme [12]. In supervised NLP, typical choice for *n* is 1-3 [13,14]. We extracted 1, 2 and 3-grams from raw, lemmatized and/or POS-tagged token classes with a predefined number of most frequent terms. During hyperparameter tuning, the number of *n*-gram terms was varied from 200 to 8000 for each token class (i.e., raw, lemmatized and POS-tags). We considered both raw counts and ratios with L2-norm TF scaling. We did not remove any functional words, such as *stop words* (e.g., ‘and’, ‘the’ or ‘or’ in English).

**2. Word embeddings:** Mikolov et al. [15] introduced *word2vec*^[[1]](#footnote-1)^, a dense vector encoding model for words. After training with a large text corpus, a word2vec model effectively captures word semantics and shows surprisingly linear relationships when trained on a large corpus. The continuous encoding efficiently produces a language model that maximizes the probability of word pairs in the same context and minimizes the probability of word pairs drawn at random from the unigram distribution. A word embedding model maps conceptual similarities of words as real-valued distances, while n-gram approach considers every word as individual with zero pairwise similarity. Word-level language model can be used to create document embeddings by averaging the word vectors. One can also apply TF-IDF scaling for the vectors before averaging [6,16,17]. When applied in supervised NLP, results depend strongly on the data and task; both good [18–20] and poor [17,18,21–24] results have been reported. Combining embeddings with *n*-grams can improve the performance [6,20]. Here, we created dense document embedding from a pre-trained 300-dimensional word2vec language model (4.2M Finnish words) and created document embeddings via TF-IDF weighted averaging.

**3. The handcrafted features:** Our model included various custom, hand-crafted features that we considered relevant for the task at hand. These included the following:

- character count (also used as scaling parameter)
- word count and sentence count
- !, ?, symbol, digit, comma and dot character count
- Overlap token count with external lists: Positive and negative word count, stop words, technical words, subjective words, dictionary words
- Number of words in the pretrained word2vec language model
- Part-of-speech (POS) tag counts: VERB, PROPN, SYM, PUNCT, NOUN, ADJ, AUX, CONJ, ADP, SCONJ, PRON, NUM
- Overlap count with common POS 2 and 3-grams
- Count of NER tags for each text

As the length of texts varied a lot, all above feature counts were also expressed as ratios (i.e., division by total character count of a text). The total number of hand-crafted features was 89 (character count+44 features+44 feature ratios). Same features were allowed to co-occur in the combined feature set. For example, symbol “?” could be also present as a unigram.

The hyperparameters related to the feature extraction included the following (with the number of combinations shown in parenthesis):

- n-gram term type: Raw, lemma and/or POS-tag (12)
- n-gram term scaling: Raw or TF scaled count (2)
- Maximum n-gram term count: Most frequent n-grams (200 to 8000 terms; total 20 steps), including all shorter n-grams (i.e., 2-grams were included in 3-grams).
- Univariate feature selection: Top response-correlated features, either global or local (50 to 1500 terms; total 5+5 steps)
- Feature scaling: Standardization or absolute maximum scaling (2)
- Learning algorithm: Six methods with method-dependent parameters (see next subsection; >6)

Our texts were in Finnish, which posed additional challenges. Finnish is a morphology-rich agglutinative language with flexible word-order, which needs to be considered in modeling [14,25,26]. In agglutinative languages, the number of word forms encountered in text is large, which results in serious data sparseness and generalizability problems when using *n*-gram approach [14,27]. Being a minority language, only a relatively small body of published tools for computational NLP exist for Finnish texts when compared to major languages, such as English. Related non-English studies include, e.g., deceptive text detection in Russian, Bulgarian and Spanish [2,8,28] and essay scoring for Portuguese [29] and German [10]. Few studies for Finnish include [30,31] for essay scoring task and [5] for topic classification.

# 2. Regression algorithms and hyperparameters

Majority of supervised NLP studies are *univariate*, i.e., each text sample is associated with a single response (e.g., score or category). Here, each text had six responses. We first created optimal models for each response independently and then used stacking - an ensemble learning technique [32] - to leverage correlations between responses and improve our predictions. Similar multivariate responses in a form of sub-scores have been previously studied for an essay and restaurant scoring tasks [29,33–35]. For example, Carvalho and coworkers [29] considered total essay scores and five *sub*-scores: Formal language, task understanding, information organization, knowing argumentation and solution proposal. Snyder and Barzilay [35] analyzed total restaurant ratings with four sub-ratings: Food, ambience, service and value. In these works, it was assumed that the total score was composed from the sub-ratings, while here each text property was considered (in principle) as independent.

We tried total six regression algorithms. In addition to the three linear regression algorithms reported in the article, we also tested the following three non-linear learning algorithms: Multi-layer perceptron [MLP; 36], Extreme gradient boosted trees [GBTree; 37] and k-Nearest-neighbors regression [NN; see, e.g., 38]. For the gradient boosting, we used implementation in XGBoost^[[2]](#footnote-2)^ library, while others were implemented in Scikit-Learn library. Best results (after hyperparameter tuning) for these models are listed in Table S1.

**Table S1.** Best non-linear model performances as measured by the *MSE ratio* (i.e., MSE of the model divided by that of a null-model) of the test set with 10-fold cross-validation. The perfect score is 0 and 1 corresponds to the null (constant) model. Each value represents the best achieved ratio after hyperparameter tuning.

| **Method** | **Trustworthiness** | **Sentiment** | **Information** | **Neutrality** | **Logic** | **Clarity** |
| --- | --- | --- | --- | --- | --- | --- |
| MLP | 0.388 | 0.367 | 0.318 | 0.440 | 0.492 | 0.489 |
| GBTree | 0.462 | 0.399 | 0.373 | 0.481 | 0.534 | 0.535 |
| kNN | 0.539 | 0.492 | 0.446 | 0.539 | 0.627 | 0.619 |

The key hyperparameters for all six (linear and non-linear) models were the following:

- Ridge: No external parameters, regularization set via internal Cross-Validation (CV)
- SVR: Epsilon margin fixed to 0.1, regularization set via internal CV
- ENet: L1/L2 ratio with values from 1/100 to 99/100, regularization set via internal CV
- MLP: 1 or 2 hidden layers with 3-50 neurons, regularization set via internal CV
- GBTree: 100-400 trees with maximum depths 2-6, regularization set via internal CV
- kNN: *k*=3, 5, 7 or 9 nearest-neighbors

For the remaining parameters not mentioned above, we used their build-in defaults. Hyperparameters were optimized internally via 7-fold cross-validation (data ratio 6:1 for training and testing) inside the training set without re-training feature extractor, i.e., the matrix *X* in the regression problem *y=*f(*X*) was not modified. While this might lead to slightly non-optimal parameters, it was deemed necessary due to high computation need of feature extractor training.

# 3. Ensemble learning models and pooling of fit coefficients

We applied bagging and stacking *ensemble learning* techniques to boost the final prediction accuracies [32] of the linear models. These two methods are illustrated in Fig. S1. While the bagging approach simply involved averaging of multiple predictions coming from different models, stacking involves multiple sequential model training stages. With six text properties, the full cycle hence contains six layers and one need to decide the order of properties (e.g., on the basis of importance). Here, in order to reduce computational load and to avoid fixing the order, we instead only used the last layer that used the predictions of independent models as additional features. In other words, for each text property, we re-trained a new prediction model that took the predicted values for five other properties. Ensemble techniques have been used previously in similar context, e.g., to combine classifiers in a sentiment analysis [39].

After fitting multiple models for a text property, we wanted to analyze fit coefficients by pooling over models. Since the models had different hyperparameters and smaller or larger feature set, typically not all features (e.g., *n*-grams) were present in all models. This required us to first convert the coefficient into a common feature space, where we simply set the missing coefficients to zero. After this transformation, we could compute and report median (or mean) coefficient over features. This “common space” transformation is illustrated in Fig. S2.


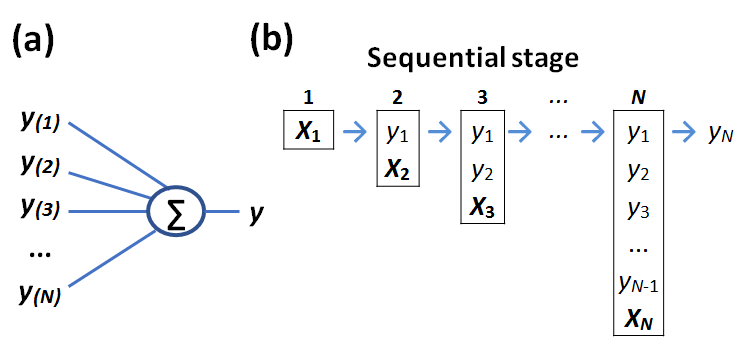


**Figure S1.** Ensemble techniques to boost prediction accuracy. **(a):** Bagging: Averaging (Σ) predictions of different models (*y*_(_*_i_*_)_) for a specific text property. **(b):** Stacking: Each prediction is added as a new feature for the next predictor. The final predictor (at stage *N*) uses predictions of all previous predictors (from *y_1_* to *y_N-1_*). Each stage represents a *different* text property.


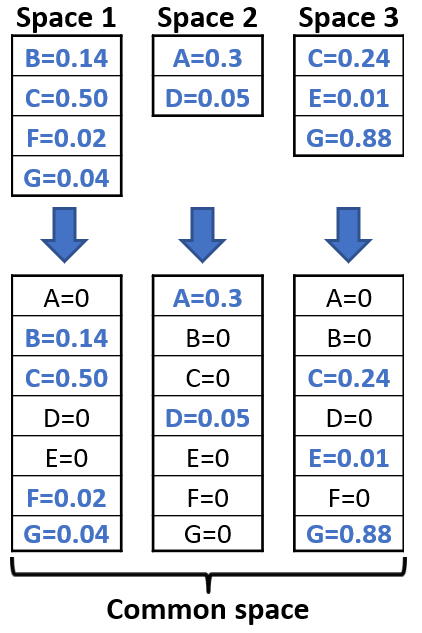


**Figure S2.** Illustration of model coefficient conversion from individual feature spaces 1-3 (with 4, 2 and 3 elements) into a common space (with 7 elements). This allows comparison of coefficient vectors related to different prediction models.

# 4. Exploratory analysis of linear fit coefficients

Here we present additional exploratory analysis of fit coefficients in the Linear Ensemble Model (LEM) that included the top 6 linear models. First, we computed the mean number of stable features and coefficient weight proportions for three feature categories. The results are listed in Table S2. The proportion indicates the fraction of total coefficient magnitude mass $\sum_{i} c_{i}$, where *c_i_* is a coefficient of a stable feature *i*. Coefficients were taken from a model trained with all data.^^[[3]](#footnote-3)^^ Along with percentages, the table also shows the relative importance of one feature, which was computed by dividing the percentage by the stable feature count (e.g., 34.6/192=0.18). The mean number of stable features was 216 for embedded features (out of maximum 300; see Materials and methods in the article), 57 for handcrafted features (out of maximum 89) and 901 for *n*-gram features (maximum not fixed, depends on hyperparameters). Total number of stable features varied between 678 and 2574 (mean 1174). On average, the main contribution resulted from *n*-gram features (66.0%), while the embedded feature had 30.7% share. Trustworthiness was an exception for both the total feature count and proportions: It had only 678 stable features in total with embedded features dominating the coefficient mass (50.2% with importance ratio 0.2). On the other hand, Sentiment had 2574 stable features from which 2260 were *n*-grams. Importance ratios for embedded and *n*-grams features were 0.09 and 0.03, which were roughly half and quarter of those for the Trustworthiness, i.e., the best models for Sentiment relied on larger array of features which were individually much weaker predictors. The remaining text properties were somewhere between the two extremes.

**Table S2.** Mean number of stable features and proportions of coefficient mass (i.e., sum over coefficient magnitudes) between three feature types. Each value is a mean over six individual linear models included in the LEM. The distribution percentages are also expressed as a ratio where the percentage is divided by the stable feature count. This is shown in parenthesis. EM = embedded features, n-grams = n-gram features, HC = hand-grafted features.

|  | **Stable features** | | | | **Weight distribution % (ratio)** | | |
| --- | --- | --- | --- | --- | --- | --- | --- |
|  | **EM** | **n-grams** | **HC** | **total** | **EM** | **n-grams** | **HC** |
| **Information** | 231 | 1063 | 75 | **1368** | 19.3% (.08) | 74.6% (.07) | 6.1% (.08) |
| **Trustworthiness** | 247 | 363 | 68 | **678** | 50.2% (.20) | 45.0% (.12) | 4.7% (.07) |
| **Sentiment** | 248 | 2260 | 67 | **2574** | 22.9% (.09) | 74.5% (.03) | 2.5% (.04) |
| **Neutrality** | 192 | 640 | 29 | **861** | 34.6% (.18) | 63.3% (.10) | 2.1% (.07) |
| **Logic** | 188 | 464 | 49 | **701** | 34.1% (.18) | 61.0% (.13) | 4.9% (.10) |
| **Clarity** | 187 | 618 | 56 | **861** | 23.1% (.12) | 71.7% (.12) | 5.2% (.09) |
| Mean | 216 | 901 | 57 | **1174** | 30.7% (.14) | 66.0% (.10) | 4.3% (.08) |

Fig. S3 depicts histograms of stable, aggregated (median) coefficients of LEMs. Coefficients were divided between three feature categories; Embedded, n-grams and handcrafted. Height of the distribution corresponds to the number of features, while the width corresponds to coefficient magnitude of the features. For linear models, larger magnitude of the coefficient is related to the higher importance of the feature.

| 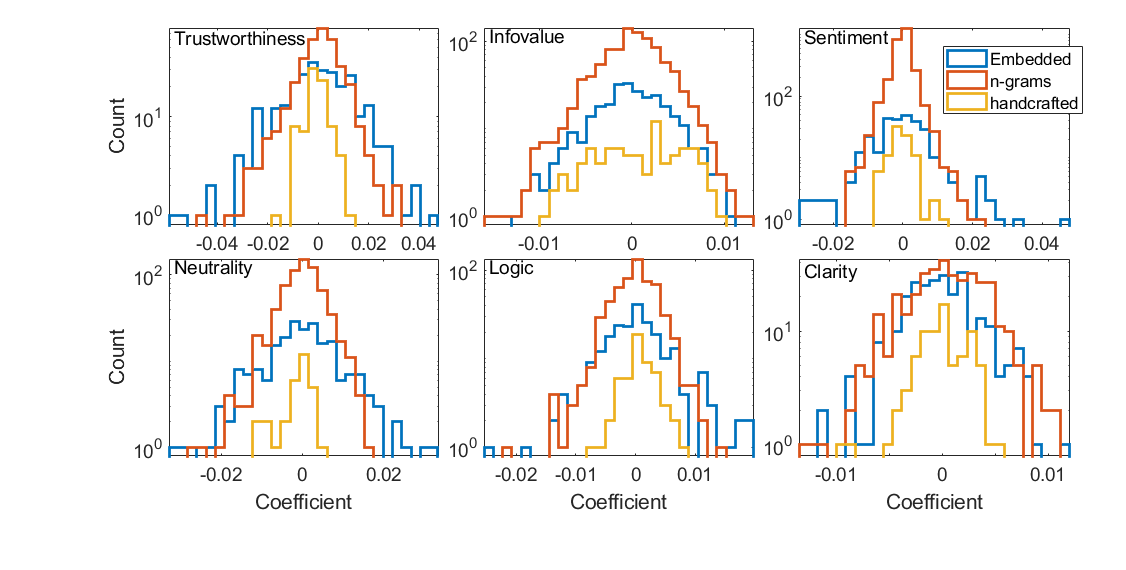 |
| --- |

**Figure S3.** Distribution of stable coefficients in the linear ensemble model. Each coefficient represents a non-zero median value over the stable coefficients of six models in LEM. Notice the logarithmic scale of the counts, which was used to allow easier inspection of the distribution shapes.

Finally, we posed the question how consistent were the individual models in LEM *between* and *within* text properties? To address this, we computed the similarity of the models both within- (*diagonal comparisons*) and between (*off-diagonal comparisons*) each text property using correlation between coefficient vectors after they were transformed into a common space. Between-property conversions were done independently for all 2^6^=64 model combinations (of the same model type) and three model types in LEM. This resulted in total 3×64=192 correlation values which were averaged. Within-property conversions were done independently for all 2^3^=8 model type combinations which were averaged. Note that because of the latter involving comparisons between model types, the two correlation types are not directly comparable, but should be only compared against other values of the same type. Correlations are listed in Table S3. Again, all correlations were positive with values between 0.16 and 0.53 for off-diagonal and between 0.42 and 0.87 for diagonal elements. Correlation between off-diagonal elements here and the ones for item biases (Fig. 4 in the article) was 0.91. As expected the model coefficient reflected the similarities of the ratings. Diagonal correlation for Information was only 0.42, which reflects some notable deviations between the weights of different models, i.e., there is more freedom in choosing the weights.

**Table S3.** Similarity of models in LEM measured by Pearson correlation of feature weights between and within text properties. Coefficients were transformed into a common space before computing correlations. Off-diagonal values represent mean correlation over all 192 model combinations (=3×2^6^), while diagonal elements represent means over 8 combinations (=2^3^).


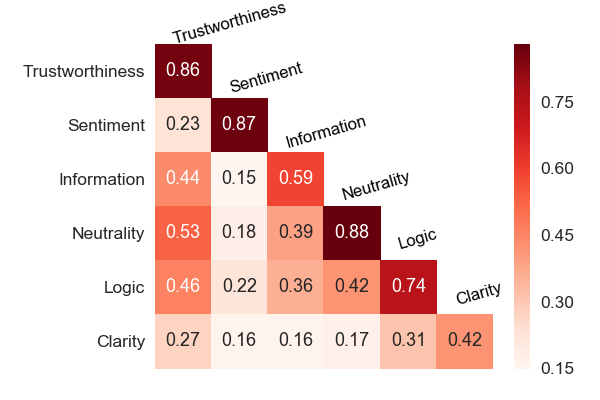


# 5. Text rating estimation and method comparison

Fig. S4 depicts Root Mean Squared Error (RMSE) of predicting ratings with 10-fold cross validation and four methods described in Materials and methods of the article. Three model-based methods result in lower RMSE than the simple arithmetic mean. Fig. S5 depicts the distributions of ratings. Note that, as the method 1 does not contain the global baseline (*µ*), we removed the global mean over ratings to make visual comparison easier. The subplots in Fig. S5 also contain the mean Pearson correlation (*r_s_*) between arithmetic mean method ratings and those given by the three model-based methods. Despite the notable differences in RMSE metric, the estimated ratings remained highly similar between the methods.

| 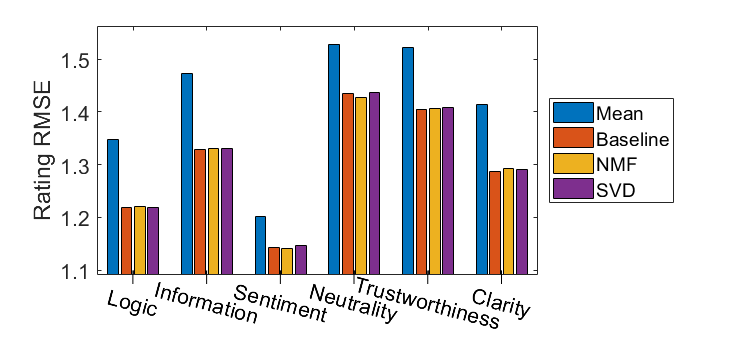 | | |
| --- | --- | --- |
| **Figure S4.** Rating prediction error (smaller values are better) for four methods with 10-fold cross-validation. For the method-based predictions, the optimal parameters were used. Model-based estimates reduce the estimation error on average by 7.8%. | | |
| 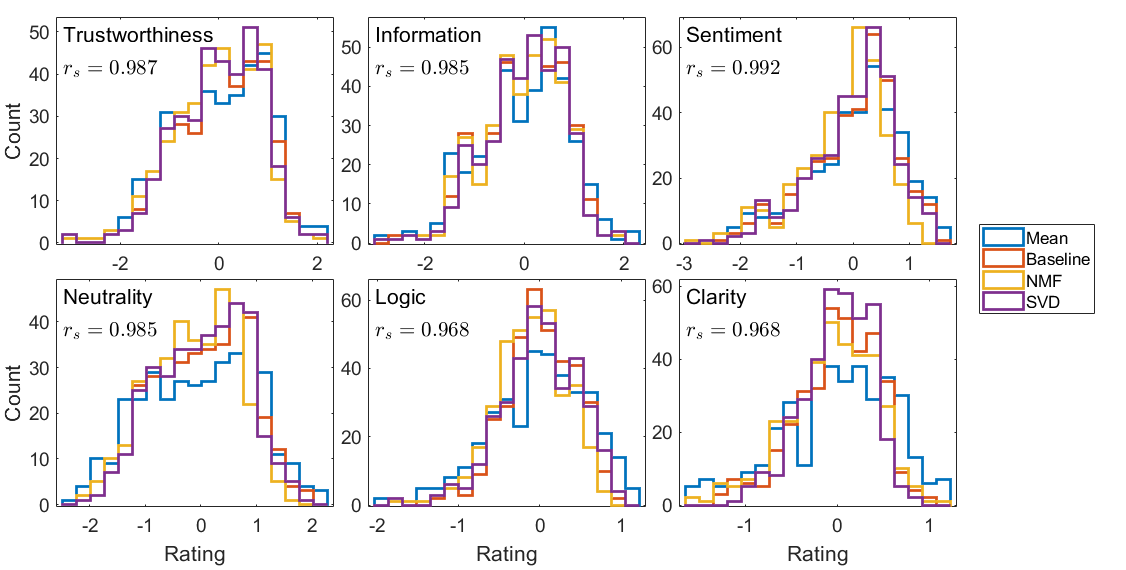 | 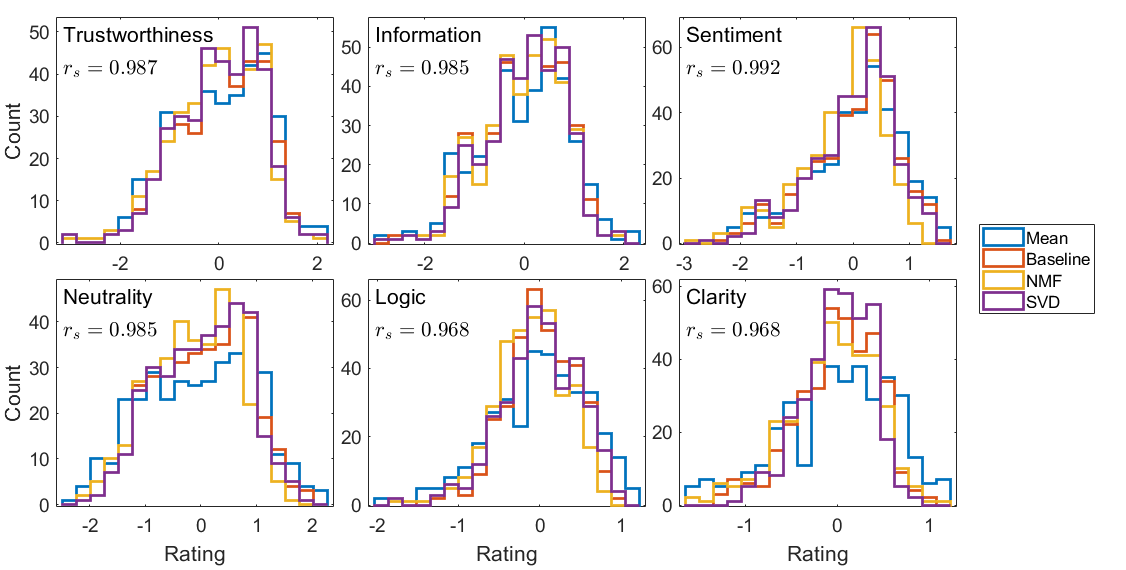 |  |
| **Figure S5.** Item rating bias histograms for simple mean and three model-based estimation algorithms. Each subplot shows rating distribution for single text property and a mean Pearson correlation (*r_s_*) between simple mean and three model-based rating estimates. These ratings are zero-centered and were the target values (responses *Y*) for the rating prediction models. They represent estimated aggregated population-level ratings and are simply here called as ratings. | |  |

# 6. Histograms of behavioral variables

Fig. S6 depict histograms of five background information variables that were collected from 407 subjects (i.e., 416 minus 9 with missing behavioral data) whose ratings were also used in computing the ratings.


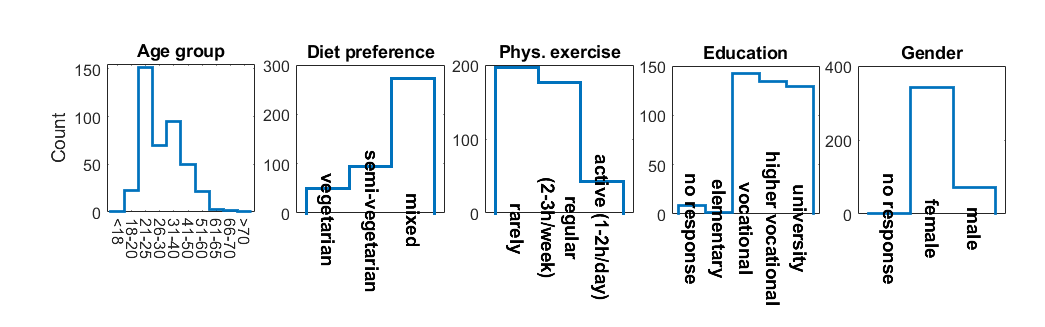


**Figure S6.** Histogram statistics of background information summary of subjects (*N*=416). Socioeconomic status (i.e., working, studying or retired) was not included as there was not enough variation for that variable (95% of subjects were working or studying).

# 7. Parameters and features of the top models

Parameters of the top predictive models (i.e., with highest mean test accuracies) are listed in Table S4. These are the external, non-trainable hyperparameters for the six models (listed in Tables 1 and S1). Pass 1 corresponds to the number of retained, most-frequent tokens and Pass 2 corresponds to feature selection using correlation.

**Table S4.** Hyperparameters of the top 4 predictive models. Here: 2g=12-grams, 3g=1-3-grams, C=term count, TF=term frequency, POS=part-of-speech, (g)=global selection, (l)=local selection, MaxAbs=Maximum absolute scaling, Std=Standardization scaling, 20+10 neurons = two hidden layers with 20 and 10 neurons, lemma=lemmatized words, raw=original words

| **Model** | **Text property** | **n-grams** | **Token type** | **Pass 1** | **Pass 2** | **Scaler** | **Other** |
| --- | --- | --- | --- | --- | --- | --- | --- |
| **MLP** | **Trustworthiness** | 3g (C+TF) | lemma | 2×200 | - | MaxAbs | 25 neurons |
|  | **Sentiment** | 3g (TF) | lemma | 1500 | - | MaxAbs | 5 neurons |
|  | **Information** | 2g (TF) | lemma+raw | 2×600 | 1500 (g) | Std | 4 neurons |
|  | **Neutrality** | 3g (C+TF) | lemma+raw | 4×300 | - | Std | 10 neurons |
|  | **Logic** | 3g (TF) | lemma+raw | 2×600 | 1000 (g) | MaxAbs | 4 neurons |
|  | **Clarity** | 3g (C+TF) | lemma+POS | 4×200 | - | Std | 20+10 neurons |
|  |  |  |  |  |  |  |  |
| **Ridge** | **Trustworthiness** | 3g (C+TF) | lemma | 2×200 | - | MaxAbs | - |
|  | **Sentiment** | 3g (TF) | lemma | 4000 | - | MaxAbs | - |
|  | **Information** | 3g (C+TF) | lemma+raw | 4×500 | - | Std | - |
|  | **Neutrality** | 3g (C+TF) | lemma+raw | 4×500 | 1500 (g) | MaxAbs | - |
|  | **Logic** | 3g (TF) | lemma+POS | 2×5000 | 2×400 (l) | Std | - |
|  | **Clarity** | 3g (TF) | lemma+POS | 2×200 | - | Std | - |
|  |  |  |  |  |  |  |  |
| **SVR** | **Trustworthiness** | 3g (TF) | lemma+raw | 2×300 | - | MaxAbs | - |
|  | **Sentiment** | 2g (TF) | lemma | 1300 | - | MaxAbs | - |
|  | **Information** | 3g (TF) | lemma+raw+POS | 3×600 | - | Std | - |
|  | **Neutrality** | 3g (C+TF) | lemma+raw | 4×400 | 1000 (g) | MaxAbs | - |
|  | **Logic** | 3g (TF) | lemma+raw | 2×600 | 1000 (g) | MaxAbs | - |
|  | **Clarity** | 3g (C+TF) | lemma+POS | 4×200 | - | Std | - |
|  |  |  |  |  |  |  |  |
| **ENet** | **Trustworthiness** | 3g (C+TF) | lemma | 2×200 | - | MaxAbs | λ=1/100 |
|  | **Sentiment** | 2g (TF) | lemma | 5000 | - | MaxAbs | λ=1/100 |
|  | **Information** | 3g (TF) | lemma+raw | 2×600 | 1500 (g) | MaxAbs | λ=1/100 |
|  | **Neutrality** | 3g (C+TF) | lemma+raw | 4×400 | 1000 (g) | MaxAbs | λ=1/100 |
|  | **Logic** | 3g (TF) | lemma+raw | 2×600 | 1000 (g) | MaxAbs | λ=1/100 |
|  | **Clarity** | 3g (TF) | raw+POS | 2×5000 | - | Std | λ=1/100 |

Table S5 lists the top-30 features with the highest weight magnitudes for Information, Sentiment and Clarity properties. Here we have omitted *n*-grams containing only stop words and averaged the weights of those features with equal *n*-grams (e.g., one in lemmatized form with TF scaling and one with raw form without scaling). The rank index indicated the actual rank of the feature when all features are included. For other three properties (Trustworthiness, Information and Logic), see Table 3 in the article.

**Table S5.** Top-30 stable aggregated non-stop word 1-3-grams and handcrafted features for LEM and three selected text properties (see Table 7 for the remaining three). Handcrafted features are marked with blue and rank shows the coefficient magnitude with respect to all features. (R) = raw term instead of lemma, (2) = average over two repeats of the same term. Symbol “**¤**” indicates paragraph change, which was considered as a token in analysis.

| **Information** | | **100×** |  | **Sentiment** | | **100×** |  | **Clarity** | | **100×** |
| --- | --- | --- | --- | --- | --- | --- | --- | --- | --- | --- |
| **Rank** | **Feature** | **Coef** |  | **Rank** | **Feature** | **Coef** |  | **Rank** | **Feature** | **Coef** |
| 1 | uskoa | -1.6 |  | 19 | se ei | 2.0 |  | 2 | VERB NOUN ADV | -1.2 |
| 2 | siihen , (R) | 1.3 |  | 23 | tietty | 1.8 |  | 4 | PUNCT NOUN NOUN | -1.2 |
| 3 | peruna | -1.3 |  | 24 | muistaa | 1.8 |  | 7 | NOUN VERB ADJ | 1.1 |
| 5 | bakteeri | 1.2 |  | 27 | suositella | -1.6 |  | 8 | VERB VERB ADV | 1.0 |
| 8 | ¤ tämä | -1.1 |  | 29 | . sama | -1.6 |  | 9 | SYMBOL_ratio | -1.0 |
| 10 | tietää , | 1.1 |  | 38 | kestää | -1.4 |  | 10 | NOUN VERB VERB | 1.0 |
| 12 | ! ¤ (2) | -1.1 |  | 40 | sellainen | 1.4 |  | 11 | PUNCT ADV NOUN | 1.0 |
| 15 | kehon (R) | -1.0 |  | 45 | mieli , | -1.3 |  | 12 | . tämä | -0.9 |
| 16 | elää | -1.0 |  | 55 | koskea | -1.2 |  | 13 | se , että | 0.9 |
| 18 | mm | -1.0 |  | 58 | tuttu | 1.2 |  | 16 | ADV NOUN | 0.9 |
| 20 | haluta | -1.0 |  | 63 | väli | -1.2 |  | 18 | ADV PRON | 0.9 |
| 21 | NEGWORD | 1.0 |  | 67 | aiheuttaa | -1.1 |  | 20 | . " | 0.9 |
| 23 | !_count | -1.0 |  | 68 | BOLDTITLE_ratio | 1.1 |  | 23 | SYMBOL | -0.8 |
| 27 | viikko | 0.9 |  | 72 | pyrkiä | 1.1 |  | 26 | toimia | -0.8 |
| 29 | huono | 0.9 |  | 76 | haitta | -1.1 |  | 32 | ravinto | -0.8 |
| 33 | minä | -0.9 |  | 80 | olla yhdistää | -1.1 |  | 33 | . | -0.8 |
| 34 | tuore | 0.9 |  | 83 | kokeilla | 1.1 |  | 34 | ( | -0.8 |
| 35 | perustua | -0.9 |  | 86 | ) . | -1.0 |  | 35 | NOUN VERB ADV | 0.8 |
| 37 | keho (2) | -0.9 |  | 89 | selvä | -1.0 |  | 37 | SCONJ NOUN VERB | 0.8 |
| 38 | ADJ_count | 0.9 |  | 91 | ei voida | 1.0 |  | 38 | ) | -0.8 |
| 39 | PRON_ratio | -0.9 |  | 96 | käyttö | -1.0 |  | 39 | ¤ NOUN VERB | 0.8 |
| 41 | !_ratio | -0.9 |  | 100 | pysyä | 1.0 |  | 44 | PUNCT VERB NOUN | -0.7 |
| 44 | hoitaa | -0.9 |  | 106 | ravintoaine | 1.0 |  | 45 | , joka olla | -0.7 |
| 46 | pystyä | -0.8 |  | 110 | hyvin . | 0.9 |  | 48 | WOMANNAME | 0.7 |
| 47 | TOTALCHARS | 0.8 |  | 114 | mm | -0.9 |  | 51 | PRON NOUN NOUN | -0.7 |
| 50 | rasva , | 0.8 |  | 117 | ORGANIZATION_tag | -0.9 |  | 54 | " , | 0.7 |
| 52 | julkaista | 0.8 |  | 120 | mainita | -0.9 |  | 55 | runsas | 0.7 |
| 55 | mainita | -0.8 |  | 122 | tuhota | -0.9 |  | 56 | ADV NOUN CONJ | 0.6 |
| 60 | COMMONPOS | 0.8 |  | 123 | olla helppo | 0.9 |  | 59 | SCONJ PRON VERB | -0.6 |
| 61 | , ei (2) | -0.8 |  | 125 | yleisesti | 0.9 |  | 61 | mahdollinen | 0.6 |

# 8. Analysis of word2vec document embeddings

Although word2vec word embeddings - nor the document embeddings derived from them - cannot be interpreted directly, one can get some insight by looking at words that are closest to document vectors in their 300-dimensional space. For this, we sorted all texts based on their Trustworthiness rating and created 10 bins, each with 34-37 texts (~10% of all 364 texts) which were averaged to get 10 document vectors (one for each bin). Then, we extracted top-20 most similar terms from the word2vec model and computed the co-occurrence of terms between all bin pairs. Results are depicted in Fig. S7. Top-20 most similar Finnish words for the highest and lowest rated bin are listed in Fig. S7a, while the word list intersection count matrix is shown in Fig. S7b. All terms were related to the theme of the texts (i.e., food and health), from which 14 (out of 39 unique) terms were mentioned (in some inflected form) in our corpus. Between the two extremes, only one term was shared (in comparison, using 1000 words, total 260 remained shared). Interestingly, terms for the lowest rated bin contain 2 commercial products (green font) and 1 misspelled word (red font; the correct word being “vehnä” or “oat” in English). These results suggest that the *least* trustworthy texts appeared being closer to informal blog texts and opinion writings rather than being carefully edited and informative content, such as news in major newspapers.

| **(a)** | **Top-20 most similar terms:** | **(b)** |
| --- | --- | --- |
| **Highest ratings**  (mean  1.30) | ei-hemiraudan (0.787),  E-vitamiinipitoisuutta, energiaravintoaineina, adiponektiiniä, puutoksille, suolinukkavaurion, lipidiaineenvaihduntaan, virtsatieongelmille, oxLDL, jodipitoisen, ravintoperäinen, elintasosairauksille, kasviskuitujen, keltarasvataudin, k-vitamiinin, valtimoille, fermentoituvien, kolesterolipitoisuuksia, insuliinineritystä, pilkkoutumattomia (0.756) | 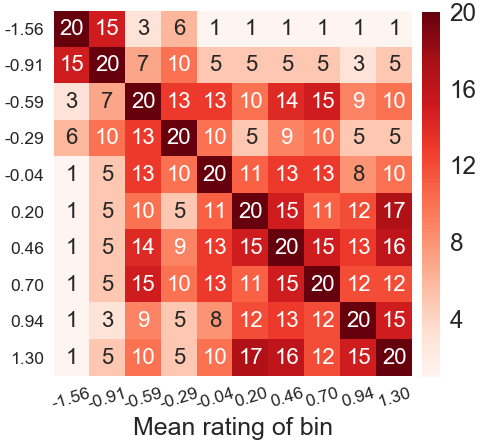 |
| **Lowest ratings**  (mean  -1.56) | Nälkäkeskuksen (0.742), viljakuituja, VitalSlimin, maitotautiin, energiaköyhempää, korkeakuituista, kuplimisena, venhä, haurastuttavan, lääkeruoan, kasvinsyöjänä, virioniin, ph-säätäjiä, ravintoaineköyhää, sunttitukos, kuparimyrkytyksen, kalkkeuttaa, nutrobal, suolinukkavaurion, homogenoimattomiin (0.698) |  |

**Figure S7.** Similarity of document embeddings obtained by averaging word2vec vectors with TF-IDF scaling. **(a):** 20 most similar words (in descending order) with an average document vector for 37 texts with the highest (top) and lowest (bottom) ratings for Trustworthiness. The similarity value is shown in parenthesis for the first and last word. Blue = shared word, Green = commercial products, Red = typo word. **(b):** Number of shared words for all 10 bins each with 34-37 texts (~10% percentile each with total count 364).

Finally, we provide English translations for terms appearing in Fig S7a. Interestingly, almost all terms were nutritional or medical related. As most Finnish terms here are morphemes or concepts, they do not have single word English counterparts. Hence, translations here correspond only to lemmatized forms of the Finnish terms and are liberal, yet allow grasping the idea.

| **Finnish word** | **English translation** |
| --- | --- |
| ei-hemiraudan | non-heme iron |
| E-vitamiinipitoisuutta | E-vitamin concentration |
| energiaravintoaineina | nutrients with energy |
| adiponektiiniä | adiponectin |
| puutoksille | deficiency |
| suolinukkavaurion | villous atrophy |
| lipidiaineenvaihduntaan | lipid metabolism |
| virtsatieongelmille | urinary tract problem/issue |
| oxLDL | oxLDL |
| jodipitoisen | containing iodine |
| ravintoperäinen | nutritional origin |
| elintasosairauksille | diseases of affluence |
| kasviskuitujen | plant fiber |
| keltarasvataudin | steatitis |
| k-vitamiinin | k-vitamin |
| valtimoille | artery |
| fermentoituvien | fermentable |
| kolesterolipitoisuuksia | cholesterol concentration |
| insuliinineritystä | insulin secretion |
| pilkkoutumattomia | non-shattering |
|  |  |
| nälkäkeskuksen | hunger center / core |
| viljakuituja | cereal fiber |
| VitalSlimin | (product name) |
| maitotautiin | milk syndrome / disease |
| energiaköyhempää | deprived of energy / low on energy |
| korkeakuituista | high with fiber |
| kuplimisena | bubbling |
| venhä | oat (note: misspelled) |
| haurastuttavan | become brittle |
| lääkeruoan | medical / medicine food |
| kasvinsyöjänä | hernivirous |
| virioniin | virion |
| ph-säätäjiä | ph-regulator |
| ravintoaineköyhää | deprived of nutrients / low on nutrients |
| sunttitukos | shunt blockage |
| kuparimyrkytyksen | copper toxicity |
| kalkkeuttaa | calcify |
| nutrobal | (product name) |
| homogenoimattomiin | non-homogenized |

# 9. Illustrations of the linear prediction process

After training a linear model, new predictions can be made by simply extracting the features, multiplying the feature vector with model coefficients and summing the resulting vector. In Figs. S8 and S9 we demonstrate this process for two example texts from the dataset. Here each token is represented by a dot and a color that indicates the weight of the feature. If there is no color, the specific token is not in the model. We also show individual prediction for the tree feature types and the top-10 n-gram and handcrafted terms with highest magnitude. Weights of features are from the LEM models (see Section S4).

| **(a)** | **(b)** | **(c)** | 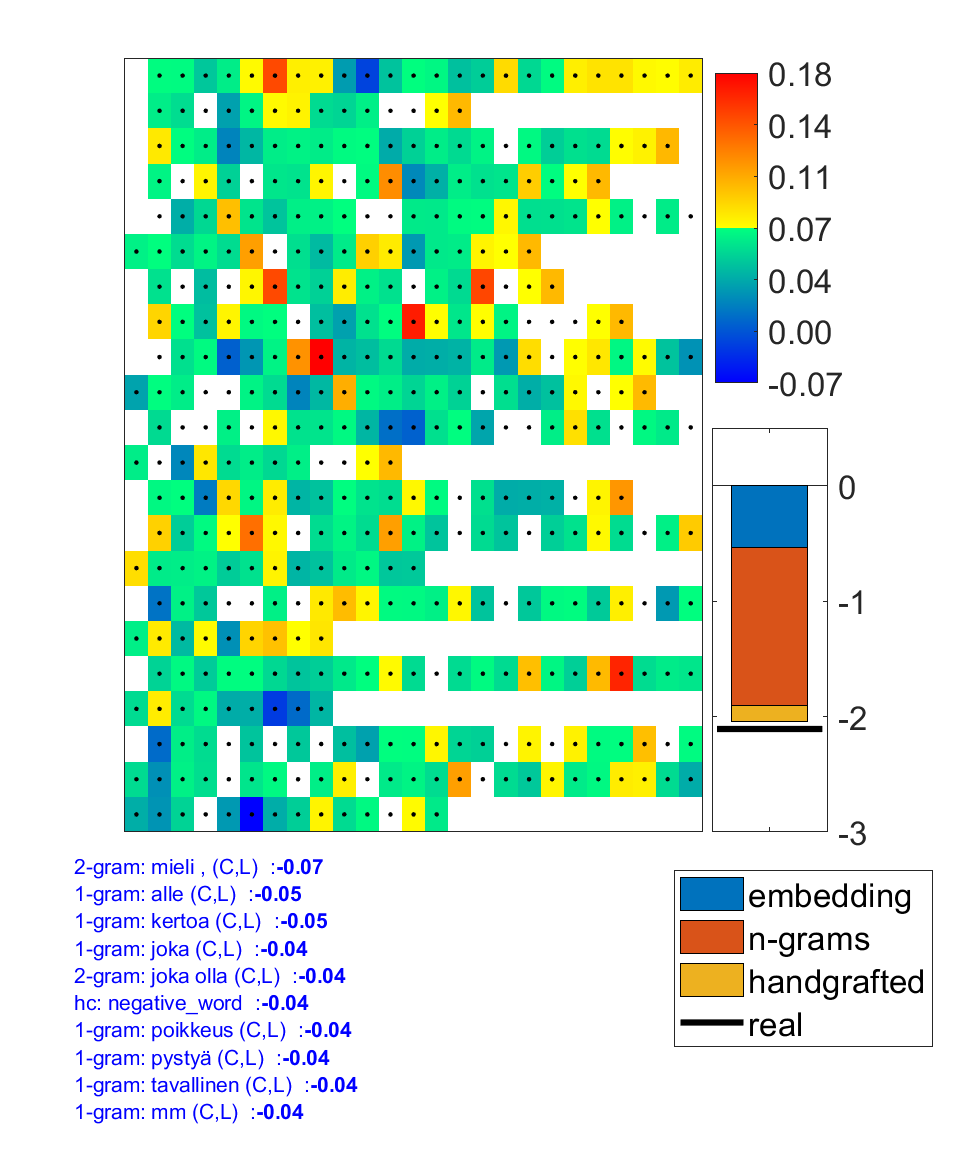 |
| --- | --- | --- | --- |
| 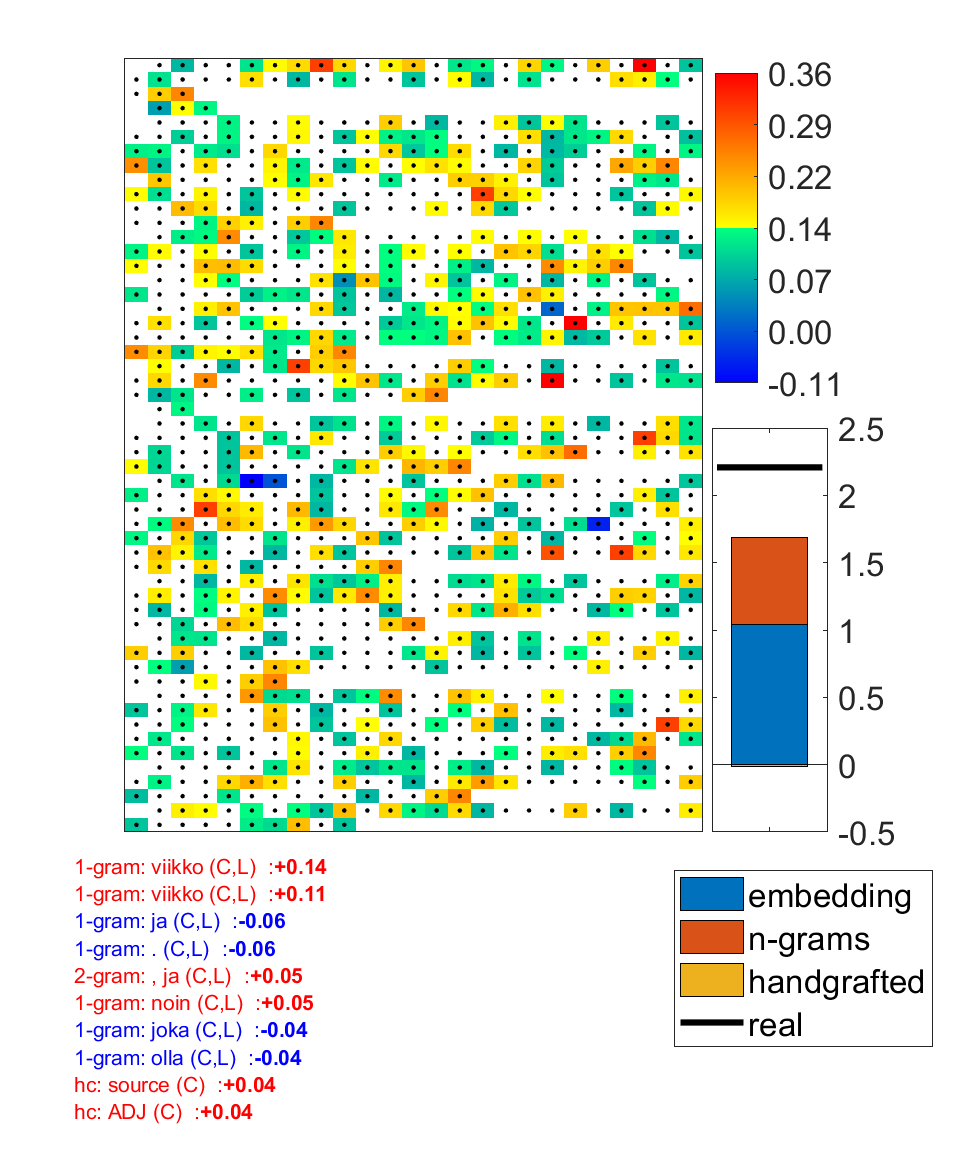  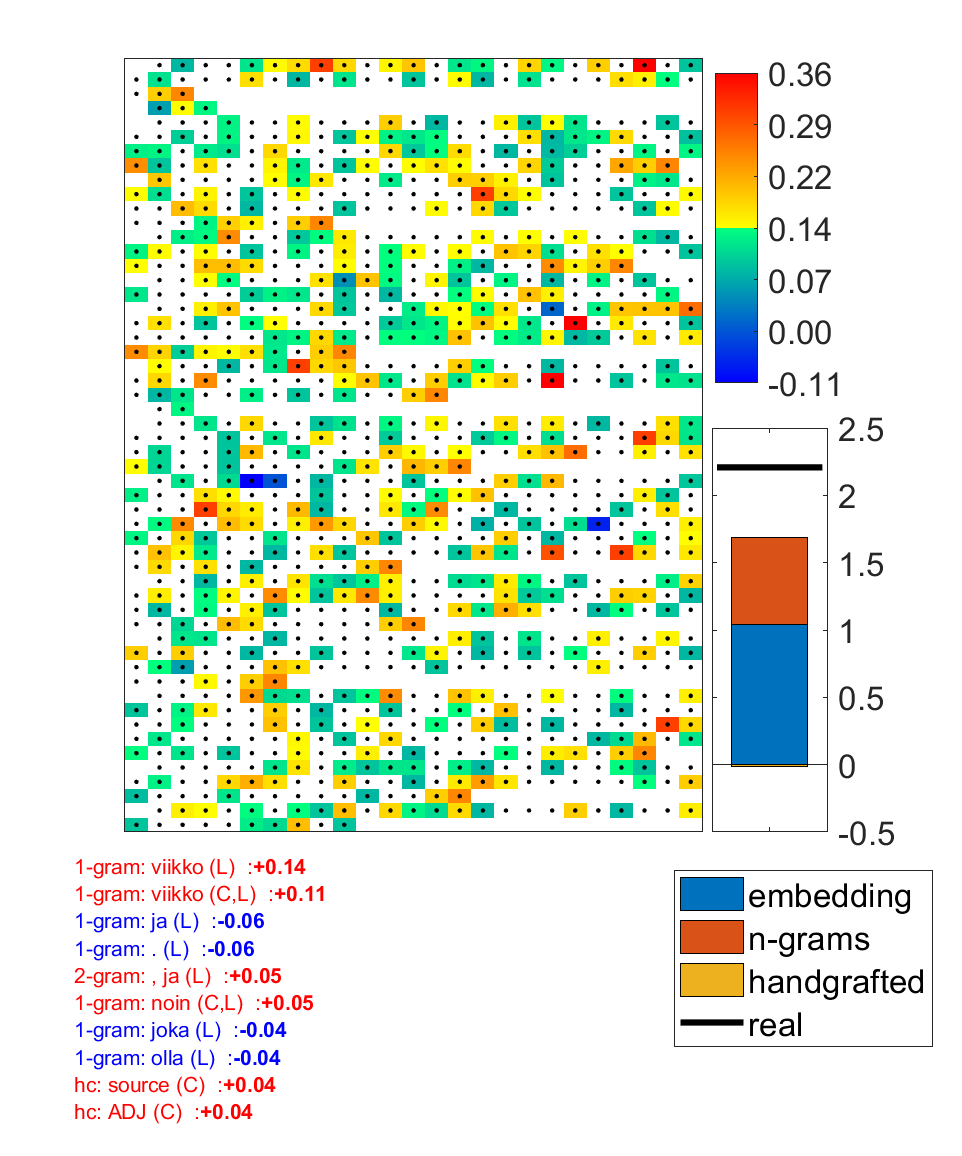 | 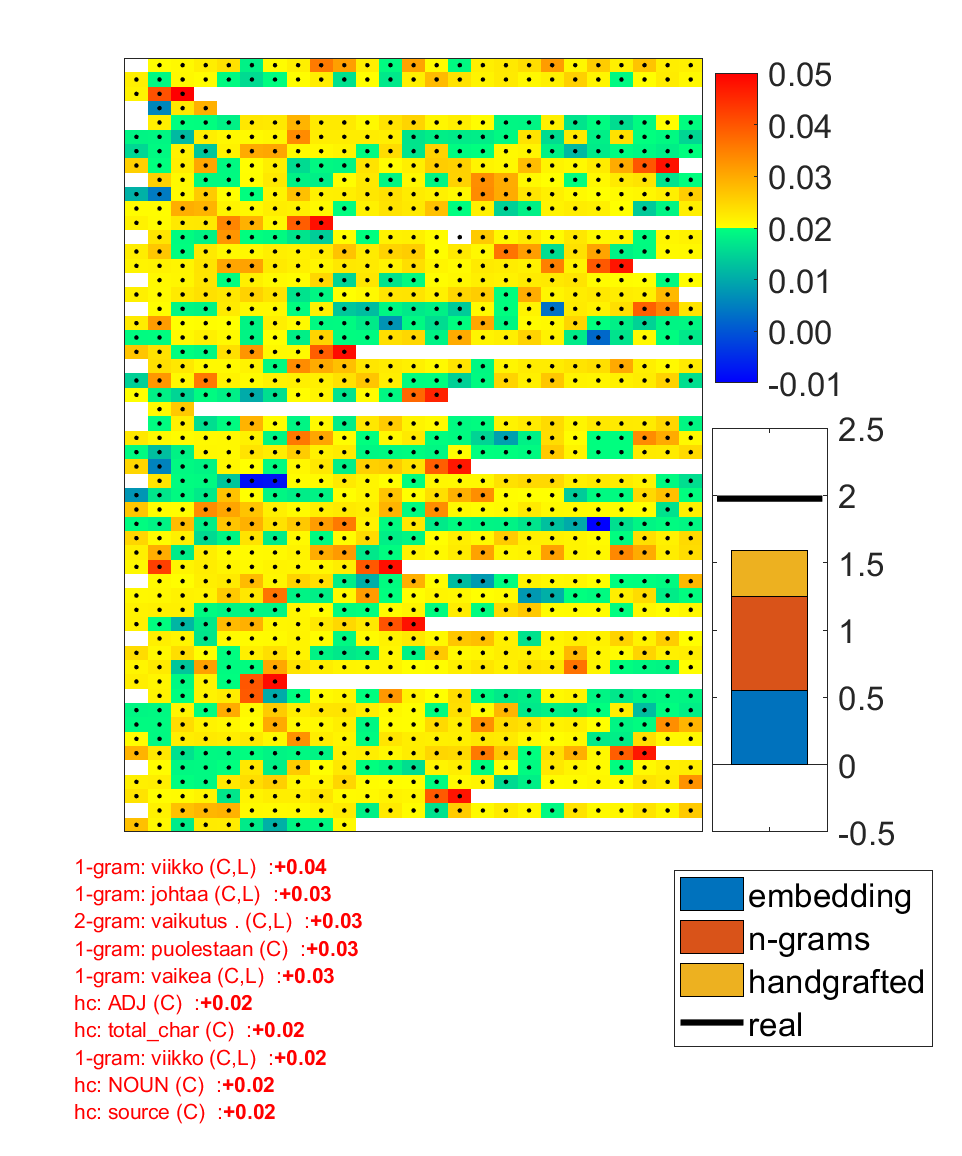  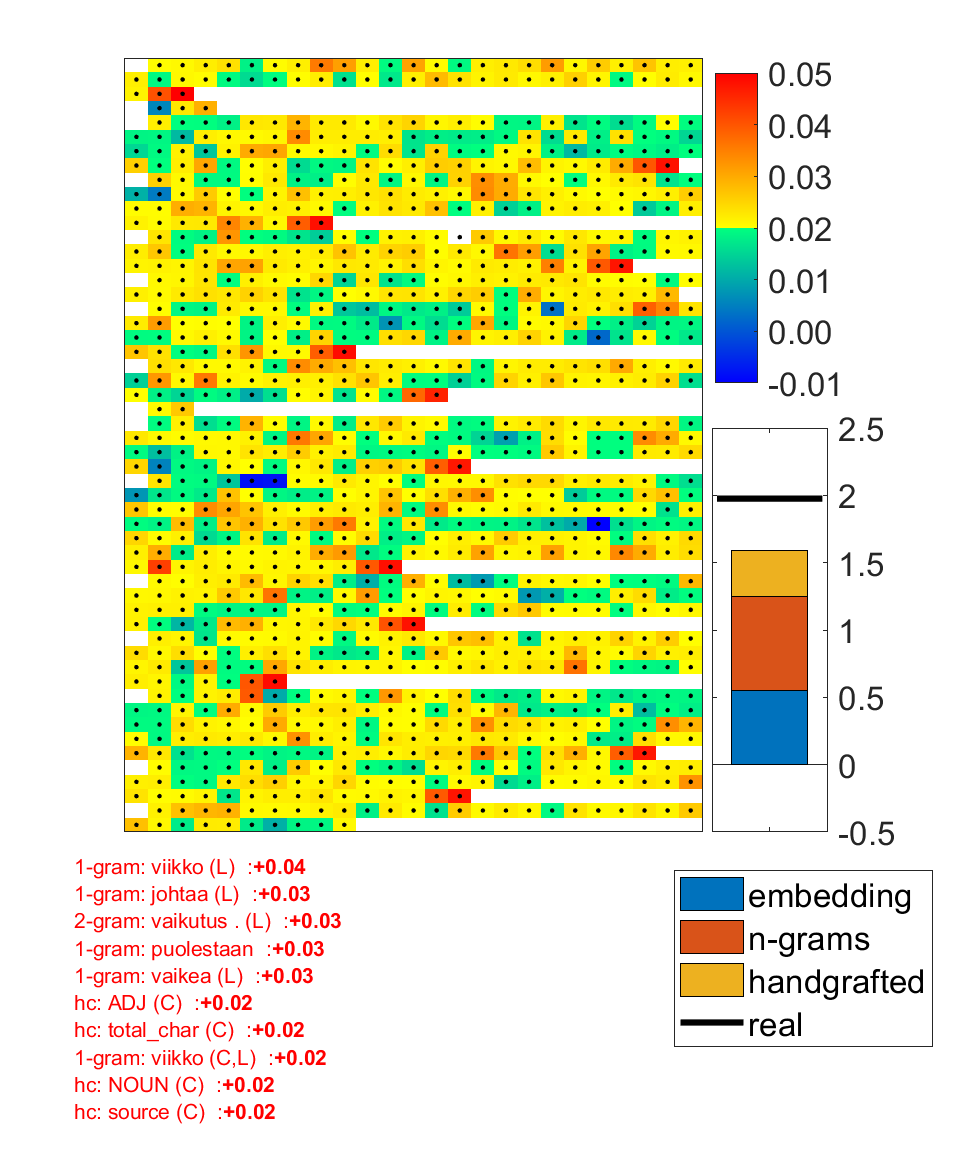 | 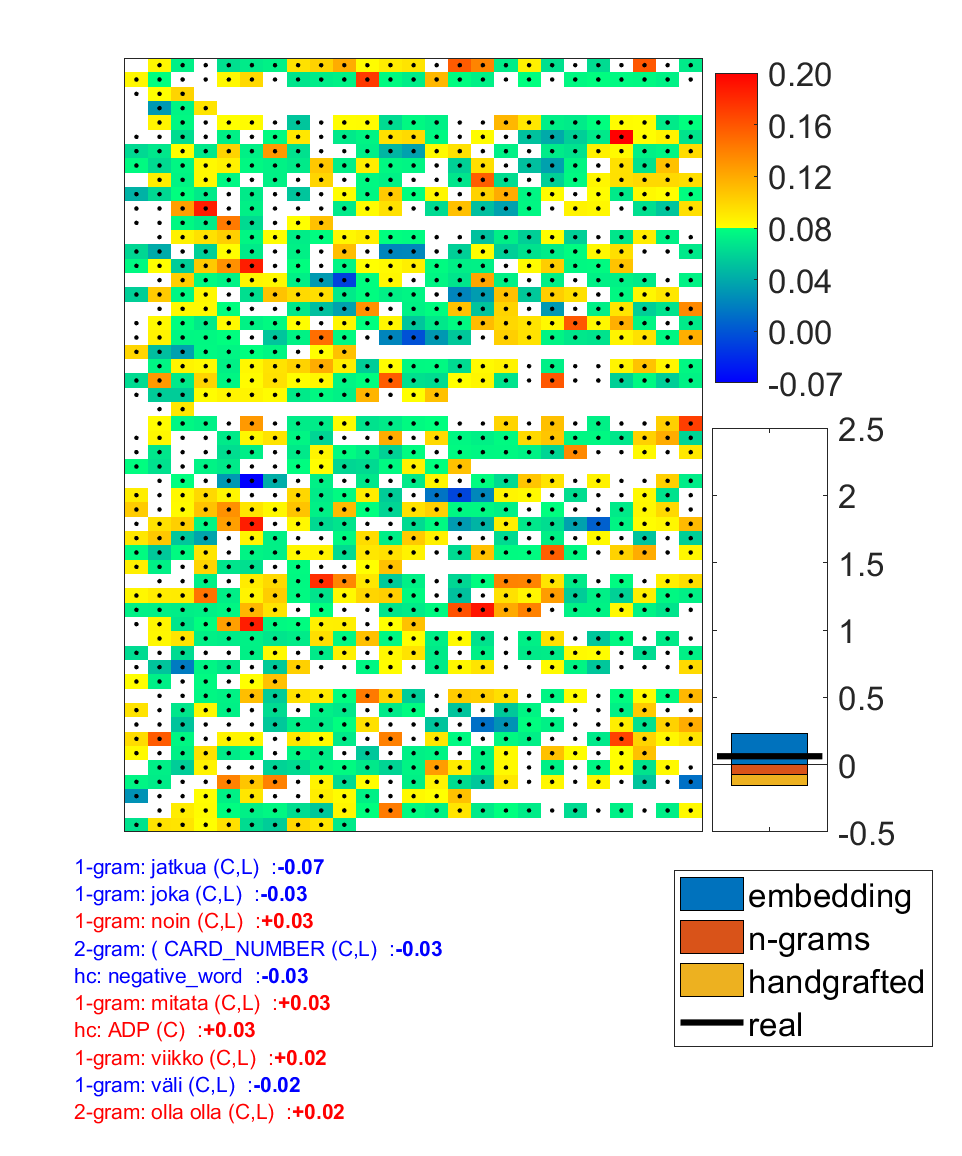  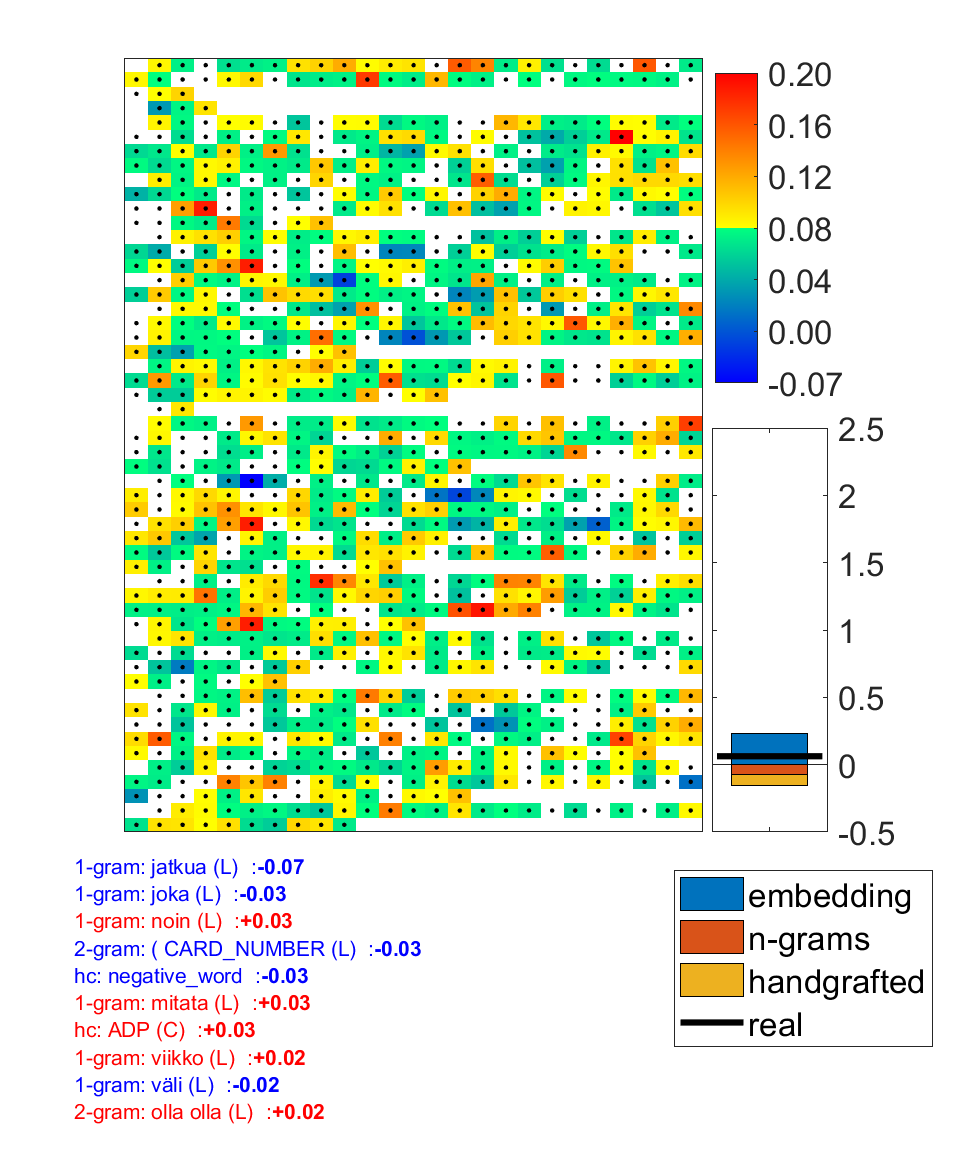 | |
| **(d)**  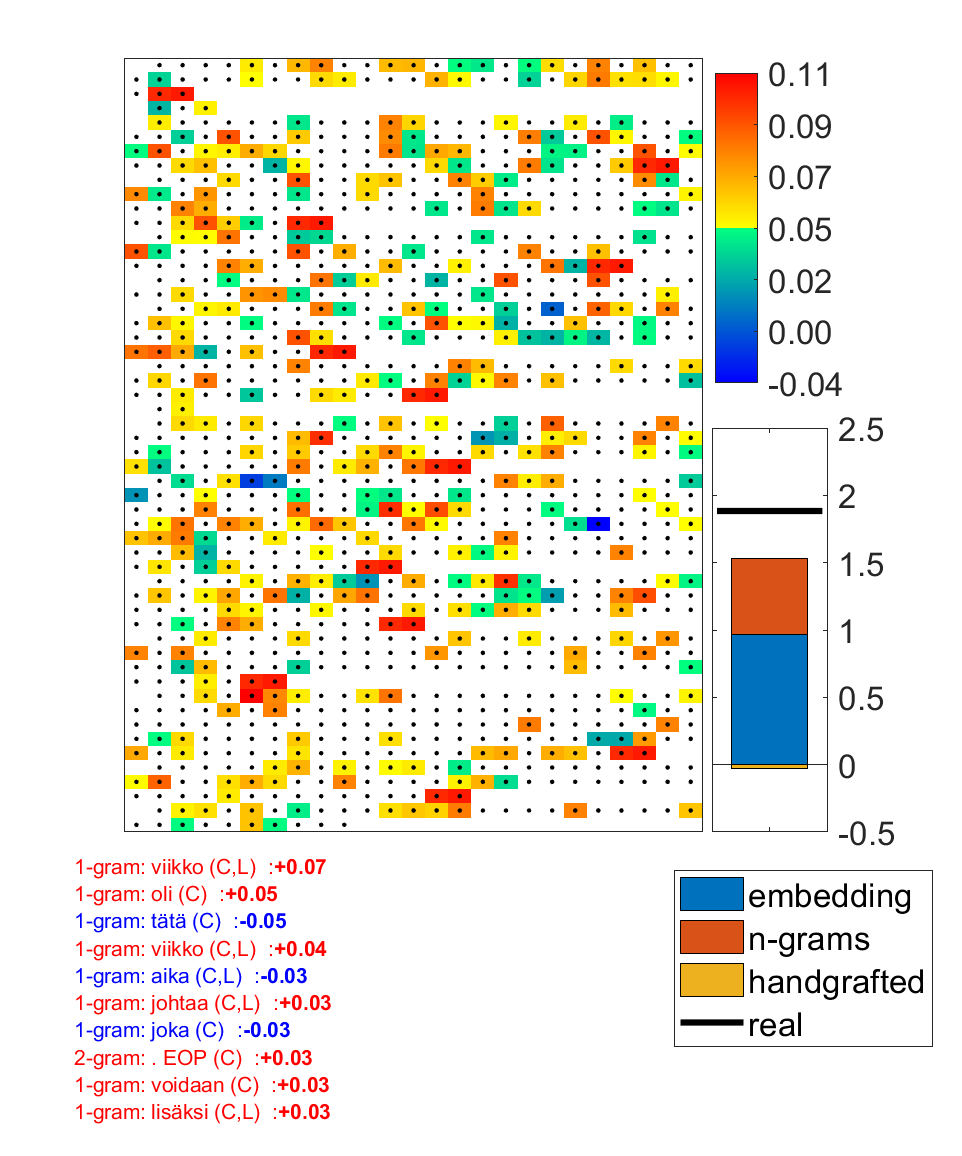  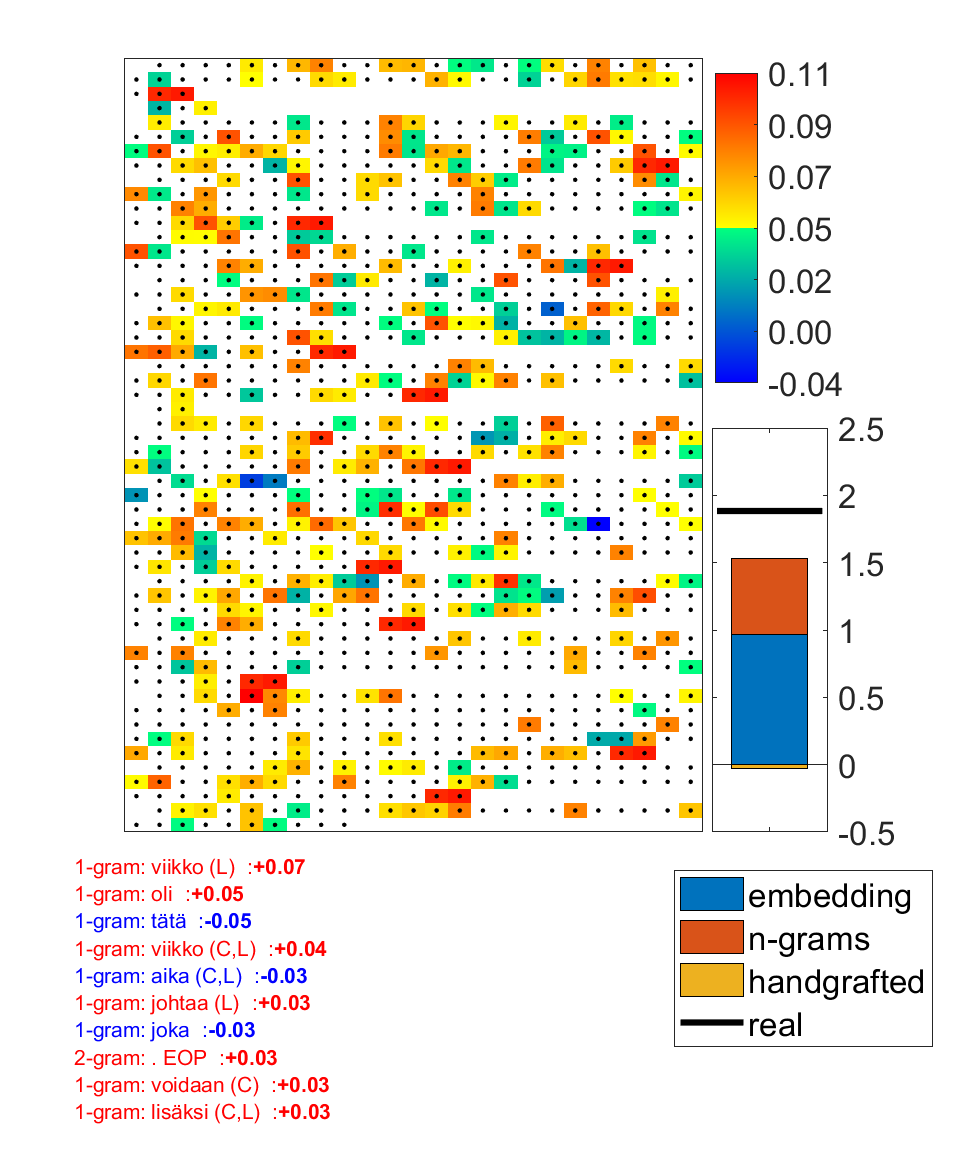 | **(e)**  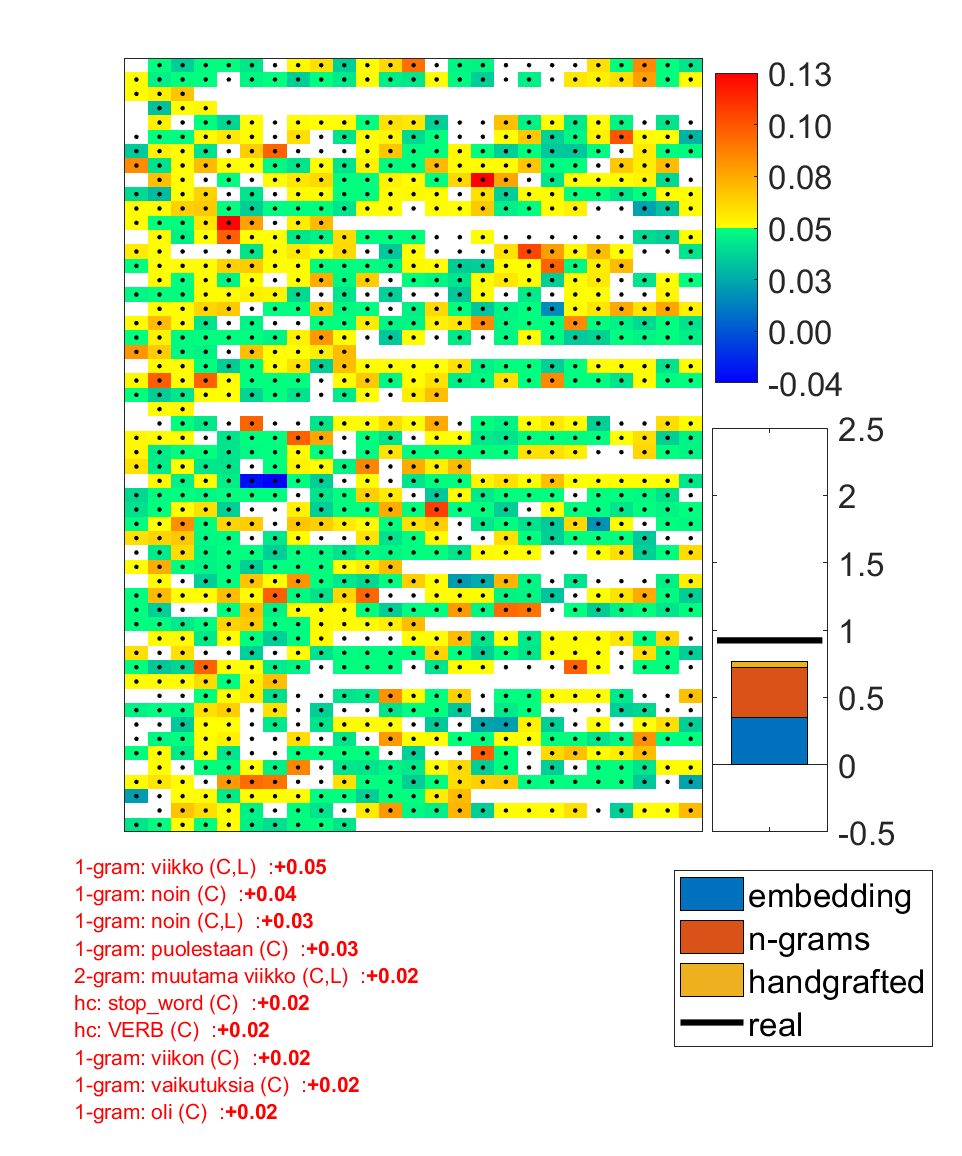  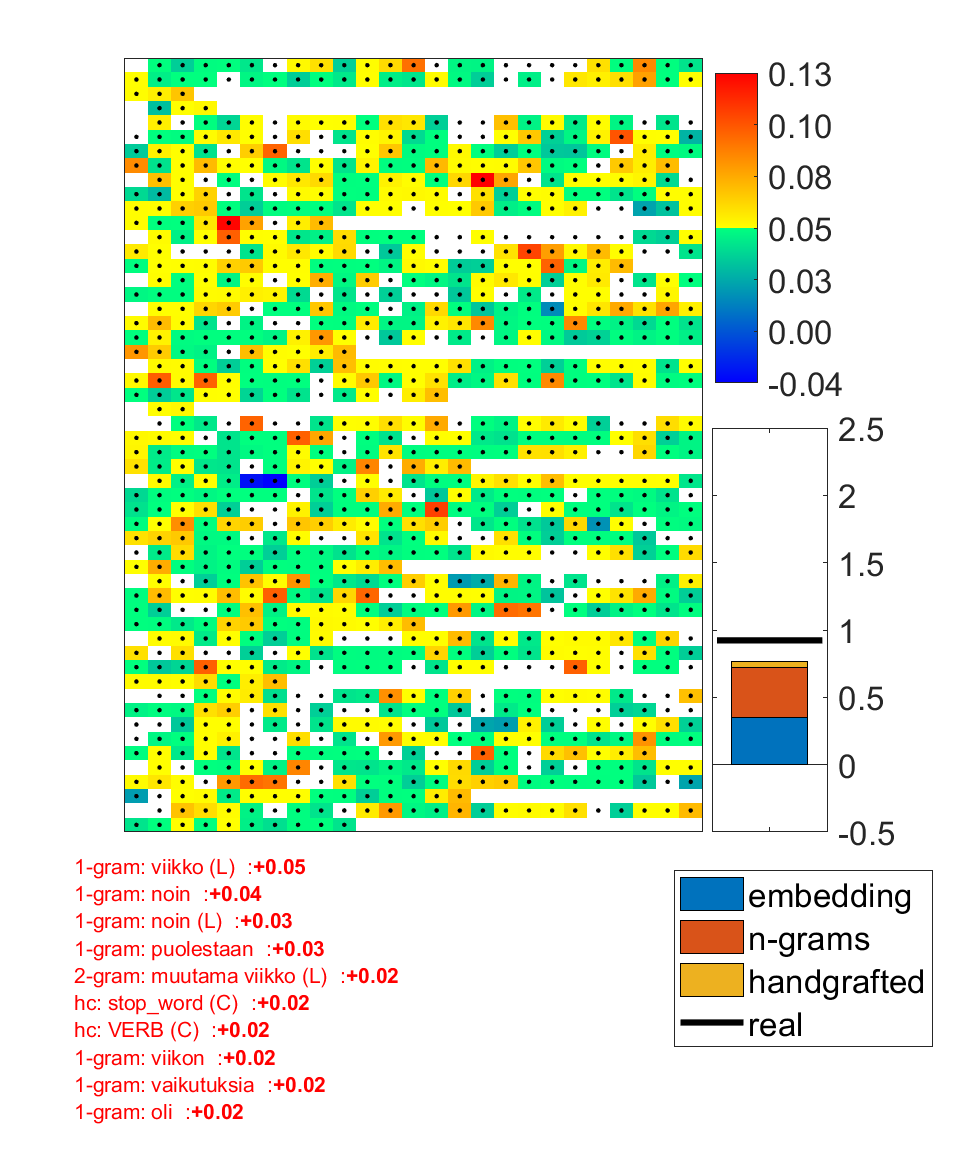 | **(f)**  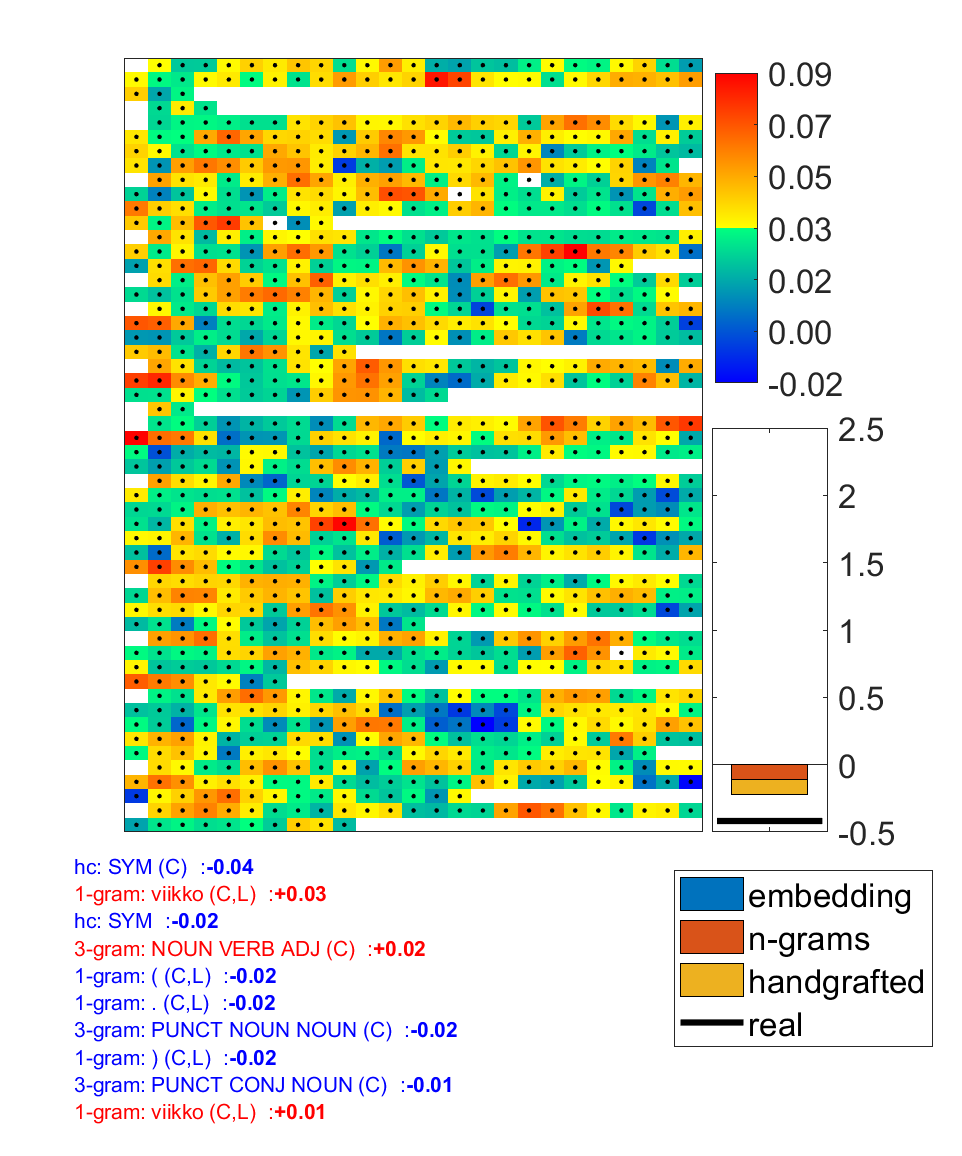  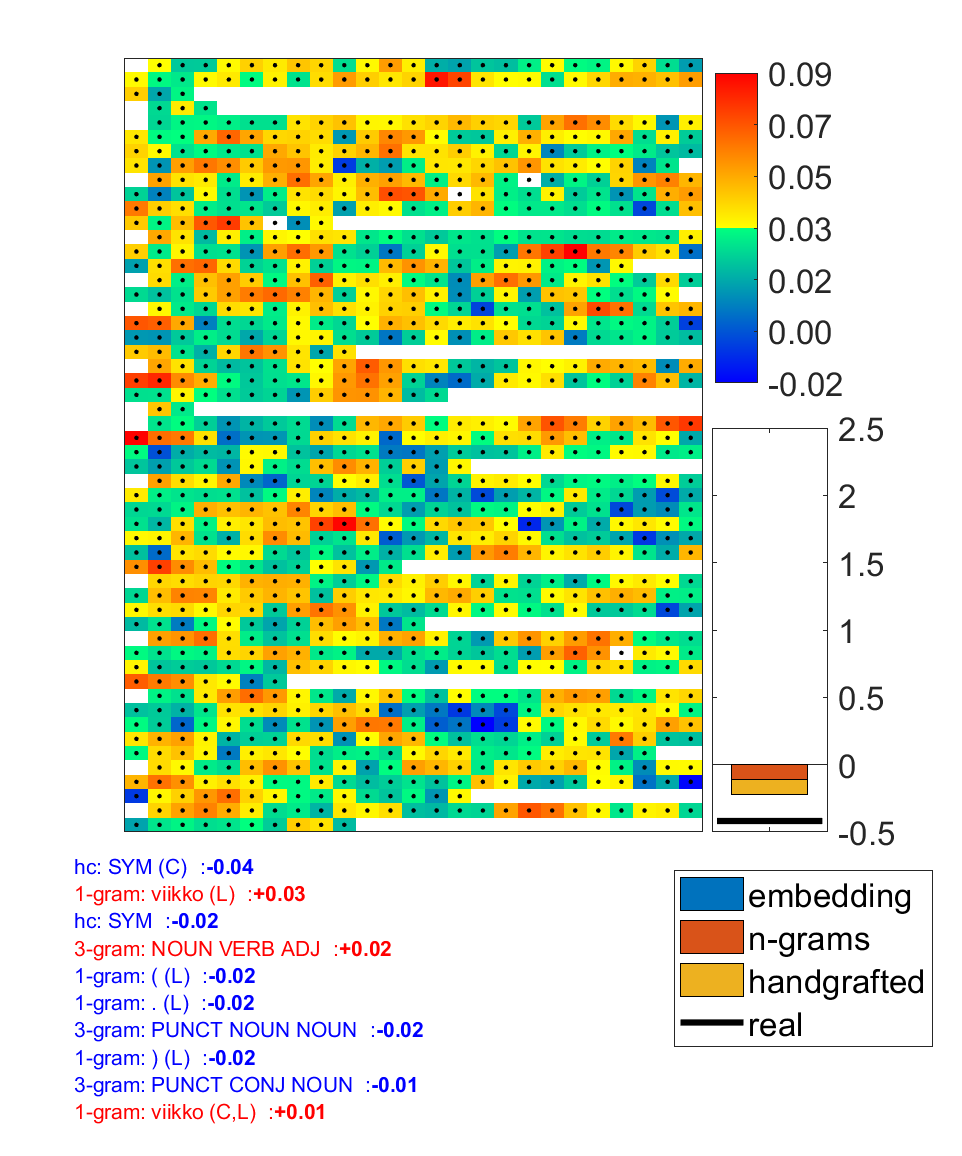 | |

**Figure S8.** Illustration of ensemble model predictions for a text sample (432 tokens) with generally *higher than average ratings*. Properties are **(a)** Trustworthiness, **(b)** Information, **(c)** Sentiment, **(d)** Neutrality, **(e)** Logic and **(f)** Clarity. Each token is represented by a dotted rectangle whose color represents the weight given to that token (positive or negative). New paragraph is marked with an empty first token. Top-10 contributing n-grams and handcrafted features are listed with the colors indicating the sign of the feature and resulting prediction for the sample (red=positive). The final prediction as a sum over the three feature types also shown with the real rating (i.e., target response *Y*). Here hc=handcrafted, L=lemma, C=count (i.e., not ratio).

| **(a)**  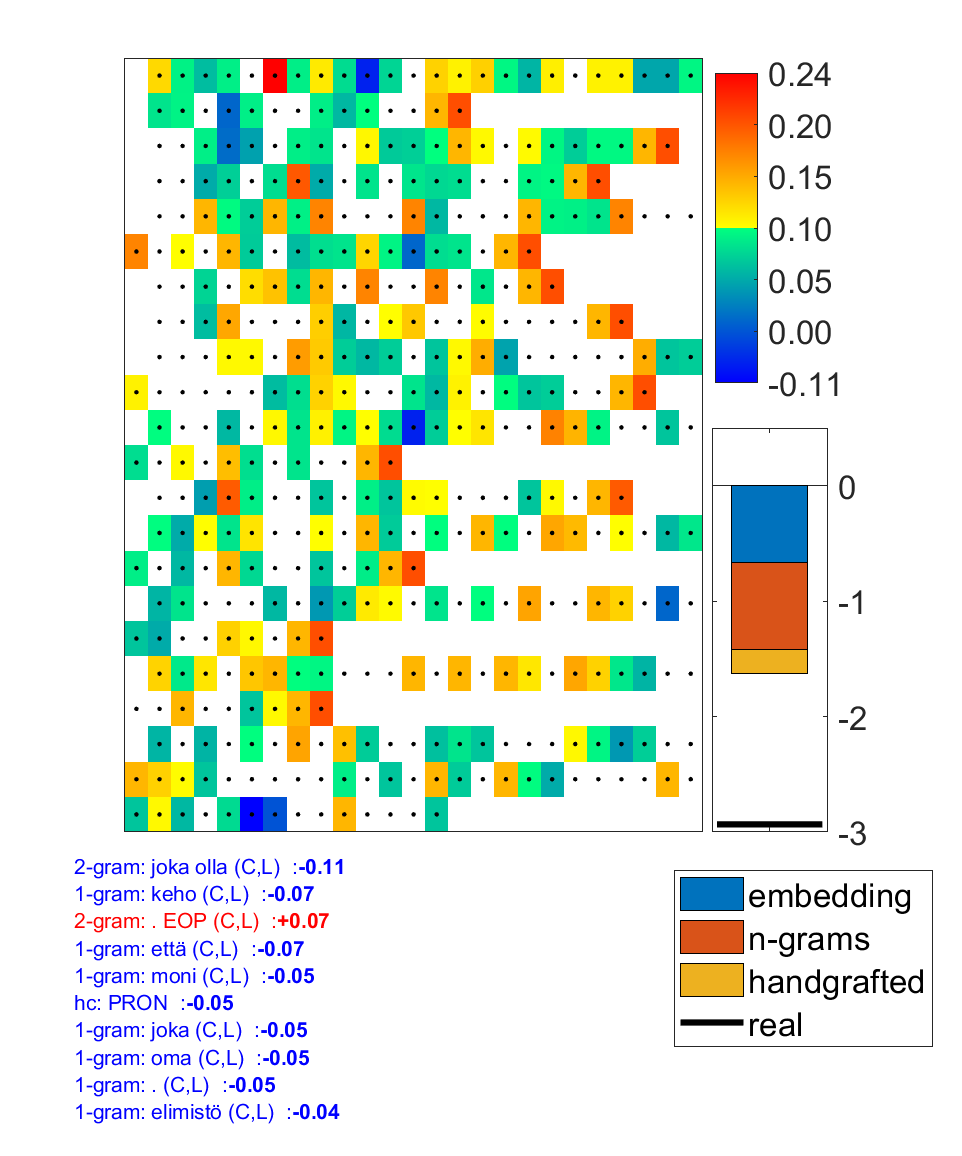  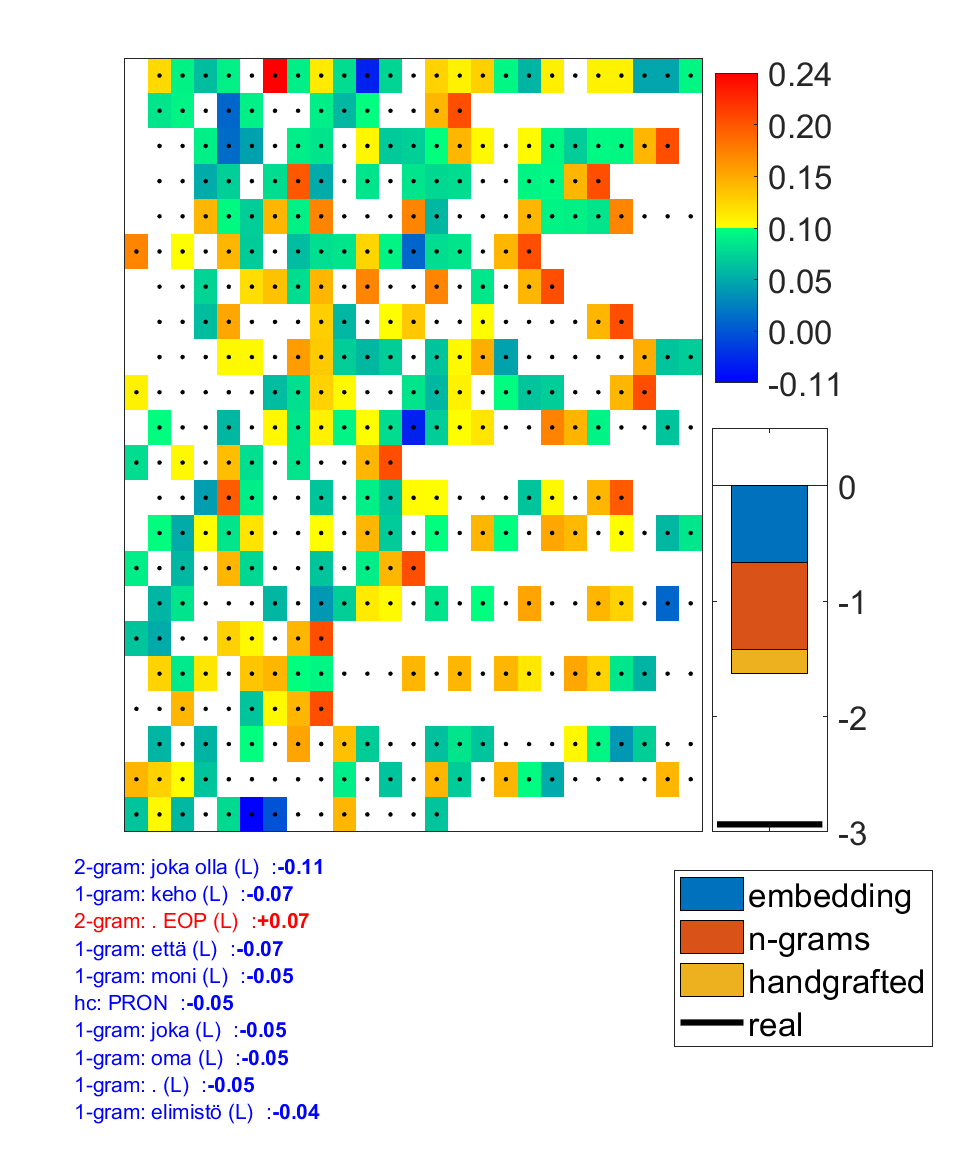 | **(b)**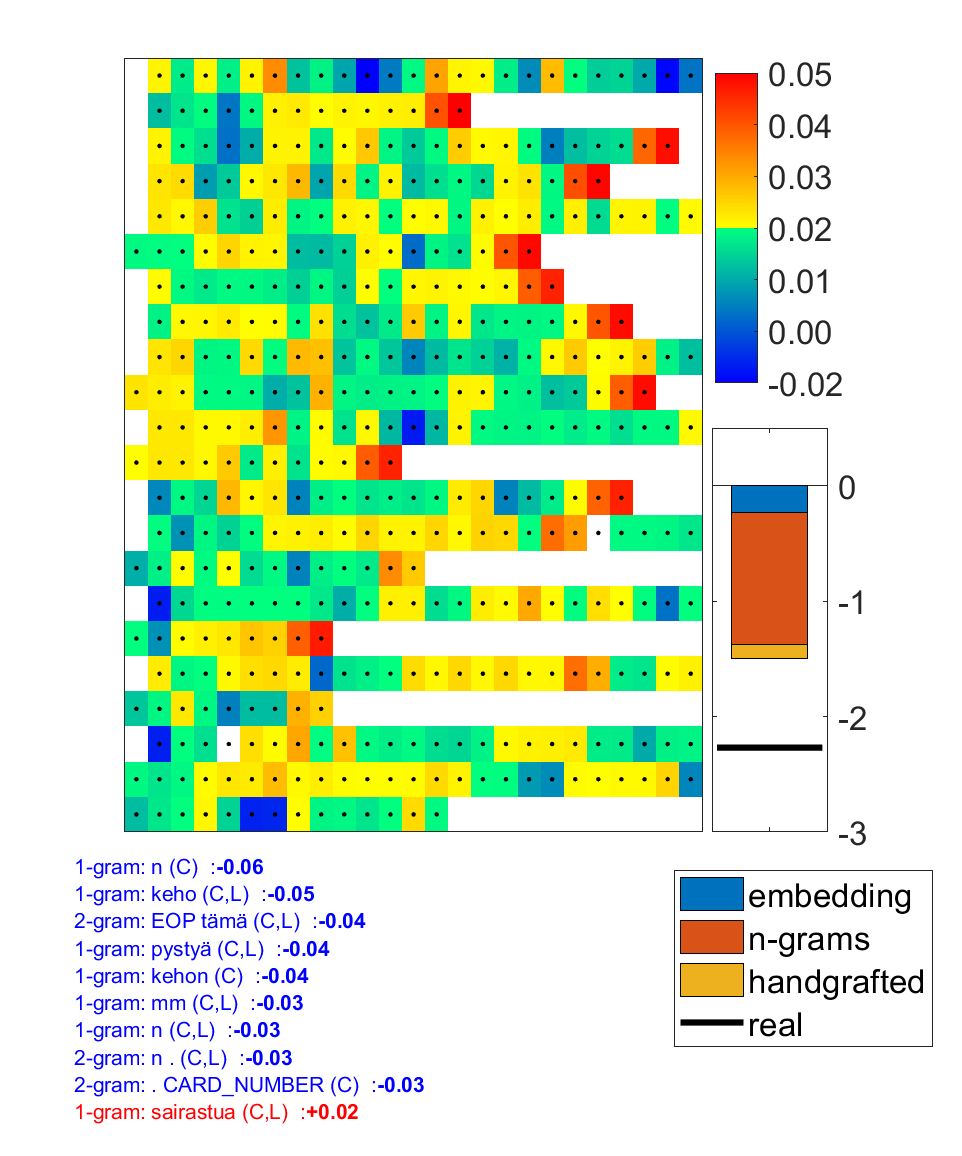  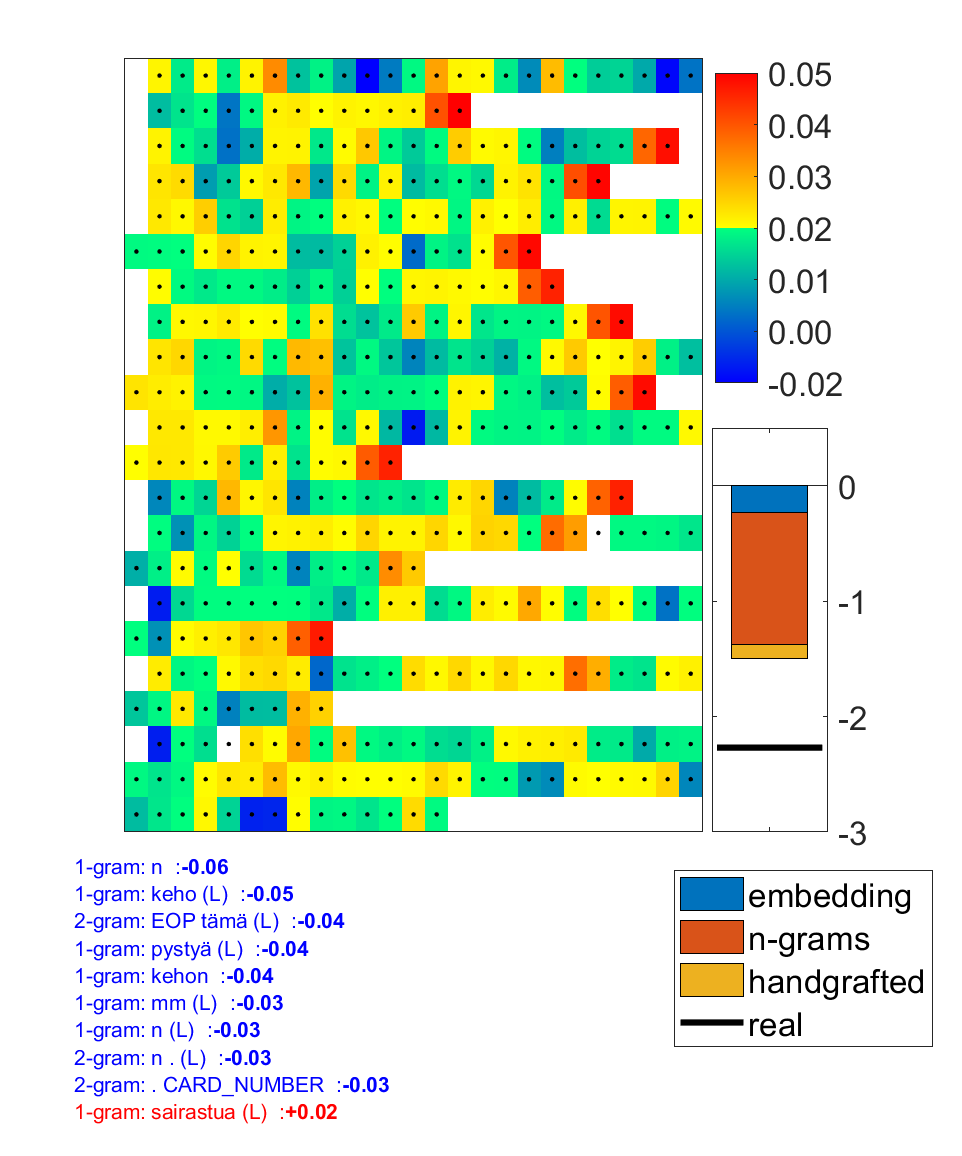 | **(c)**  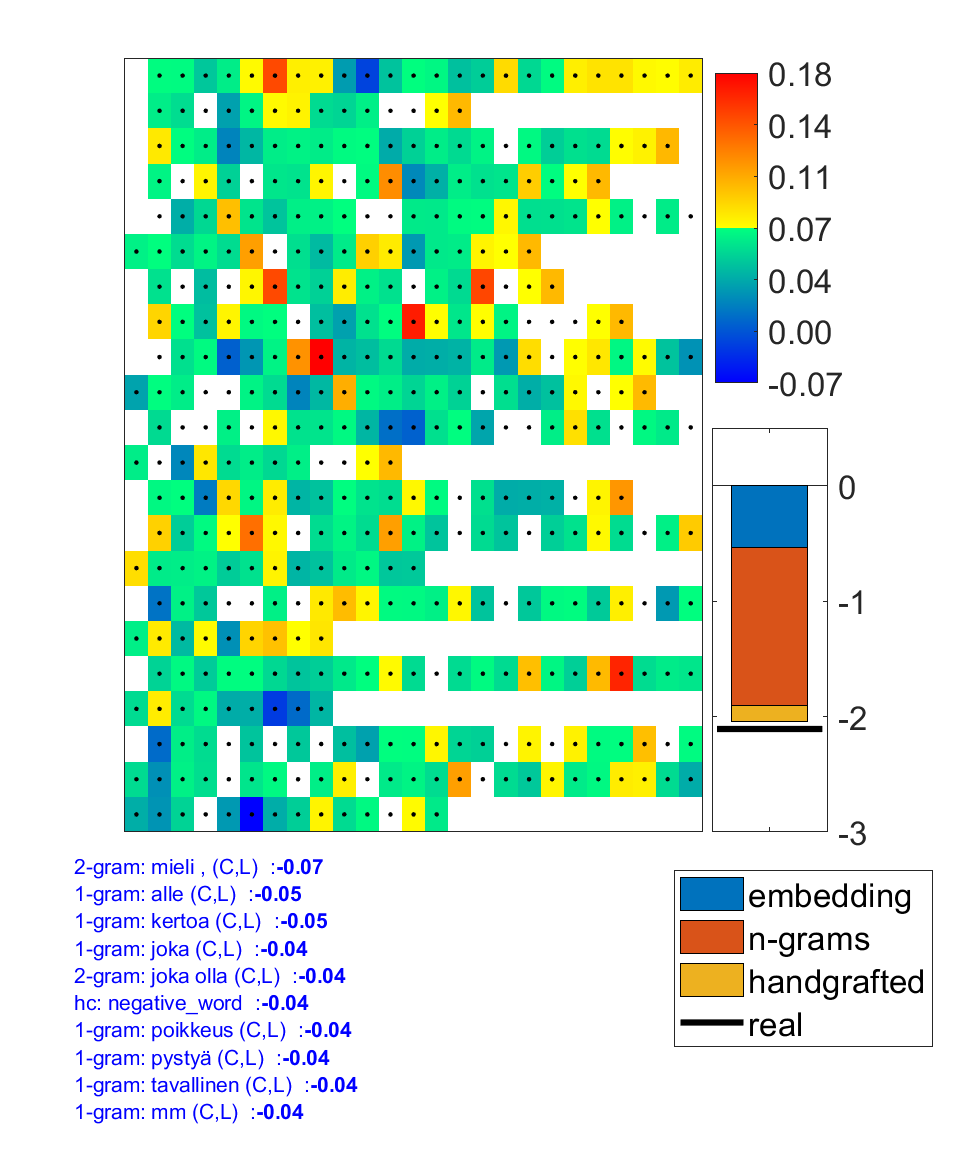  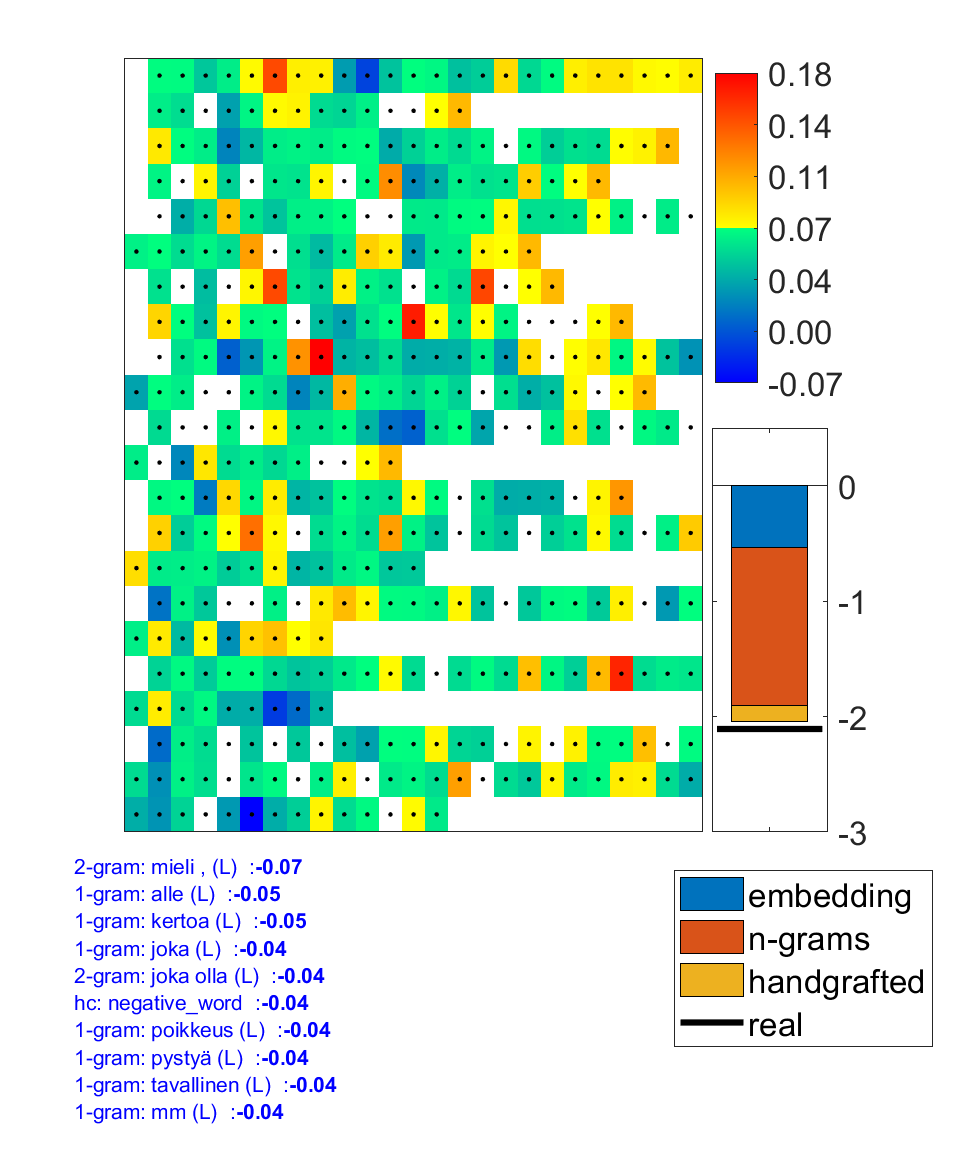 |
| --- | --- | --- |
| **(d)**  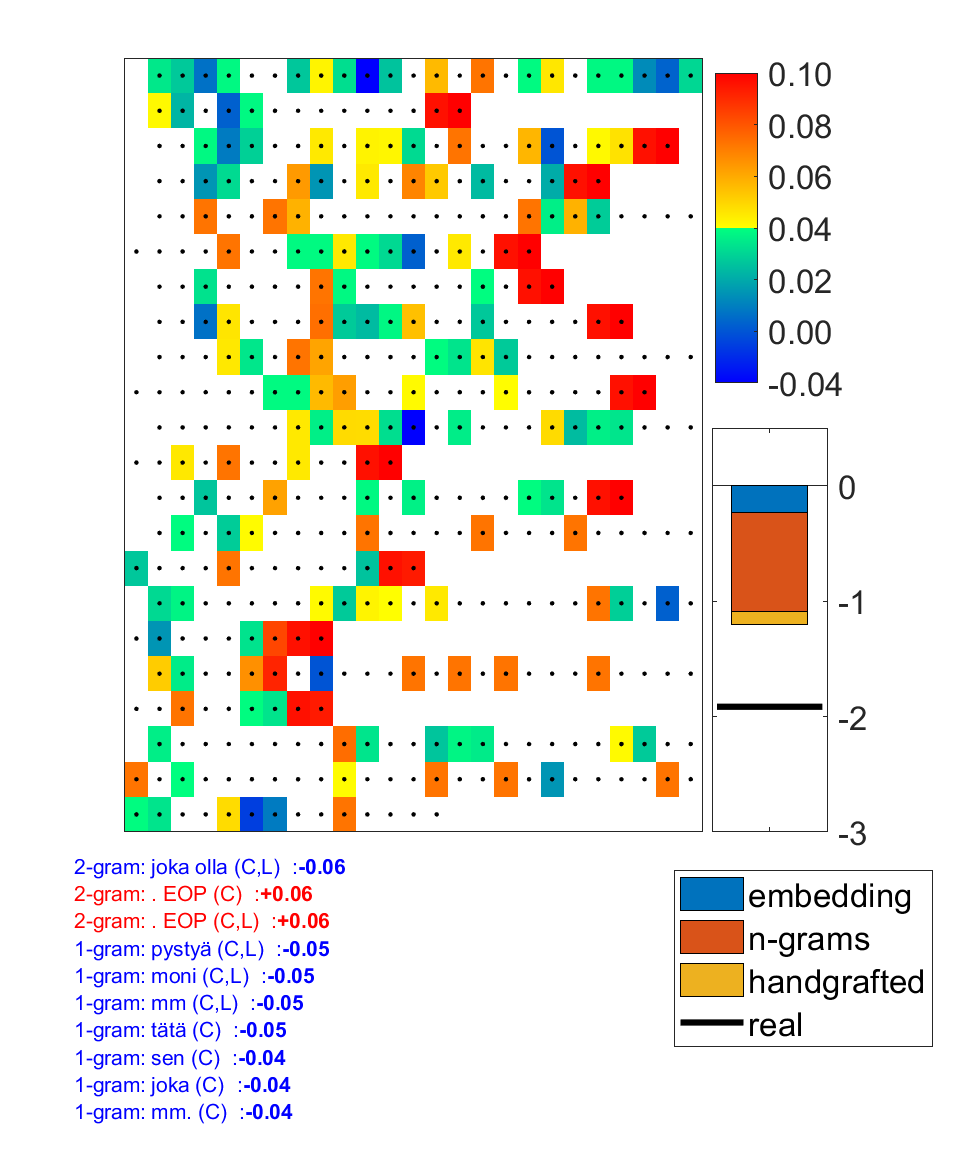  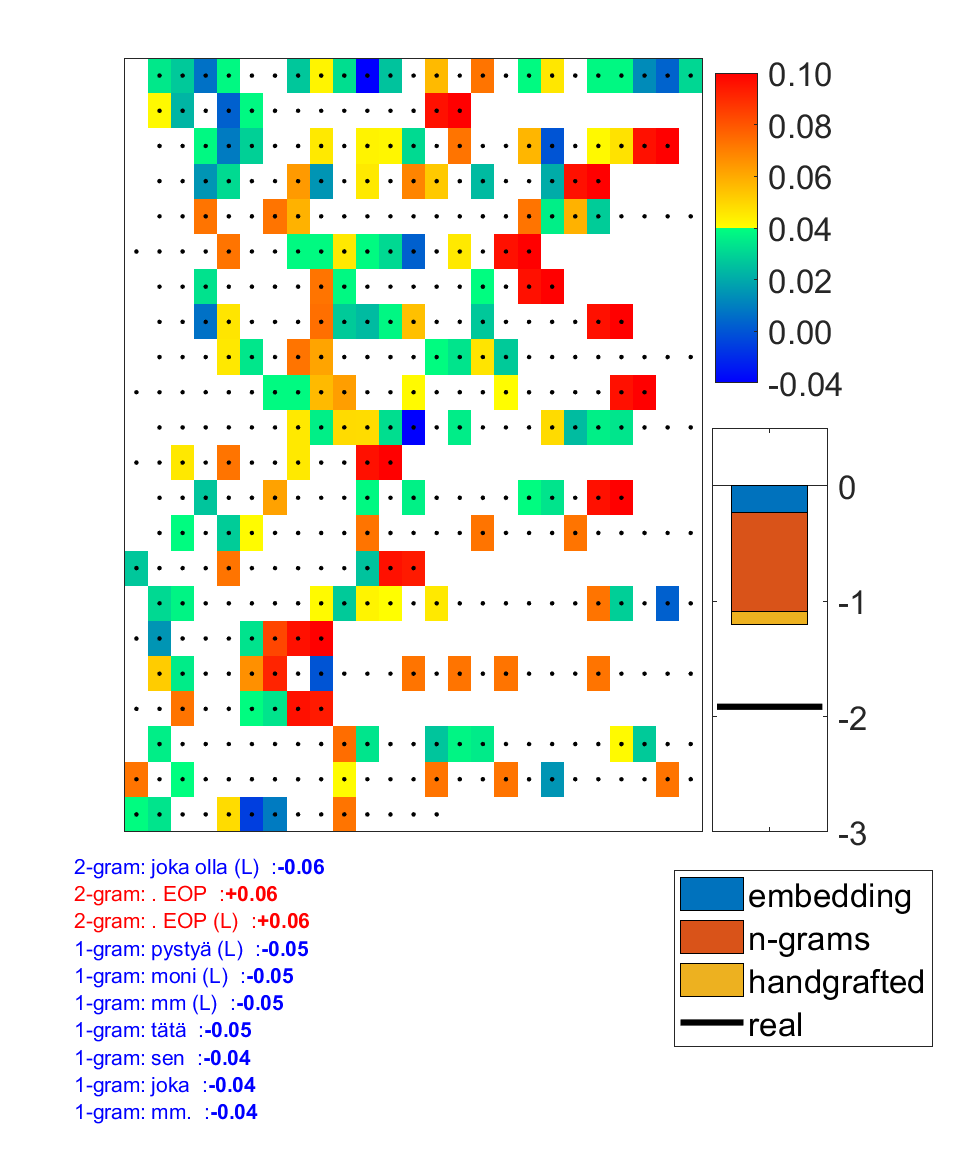 | **(e)**  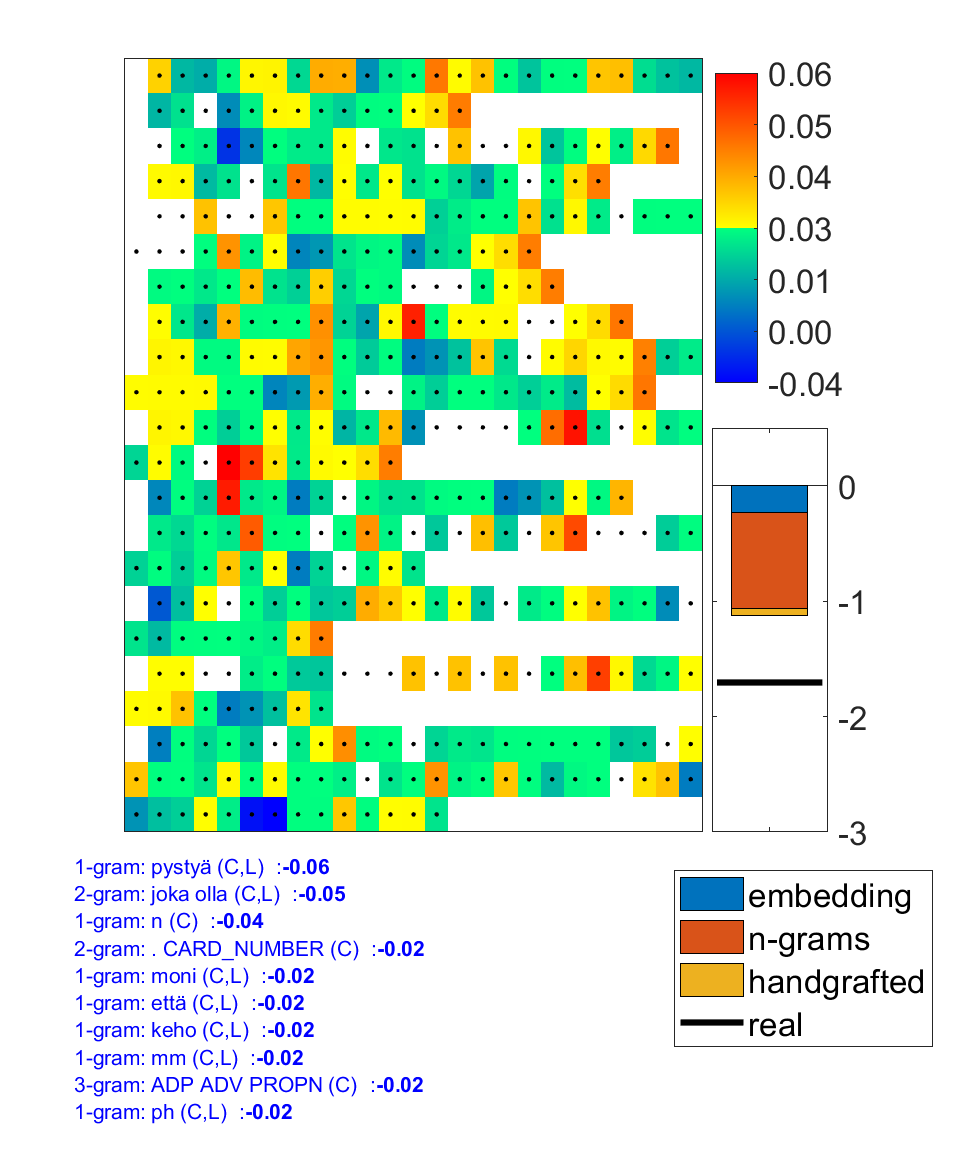  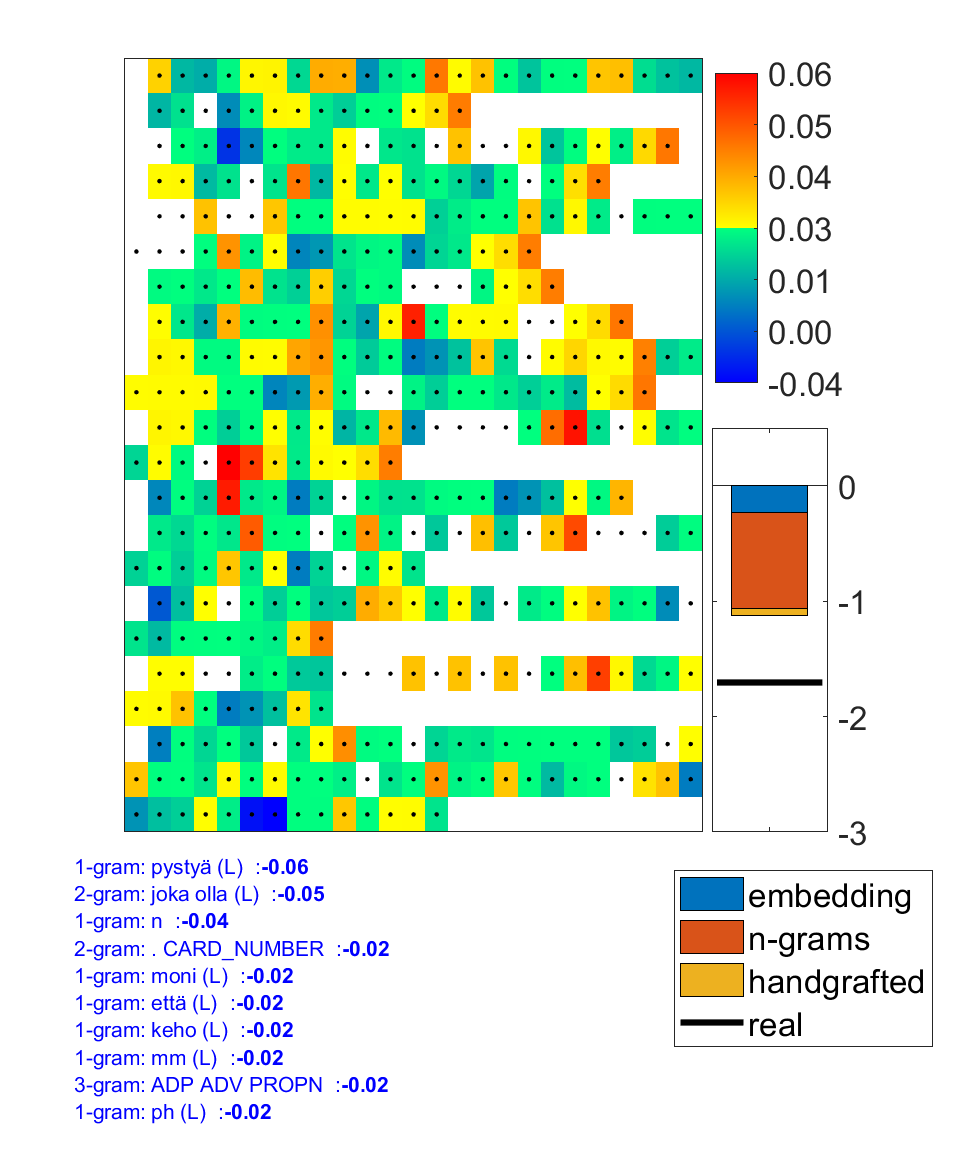 | **(f)**  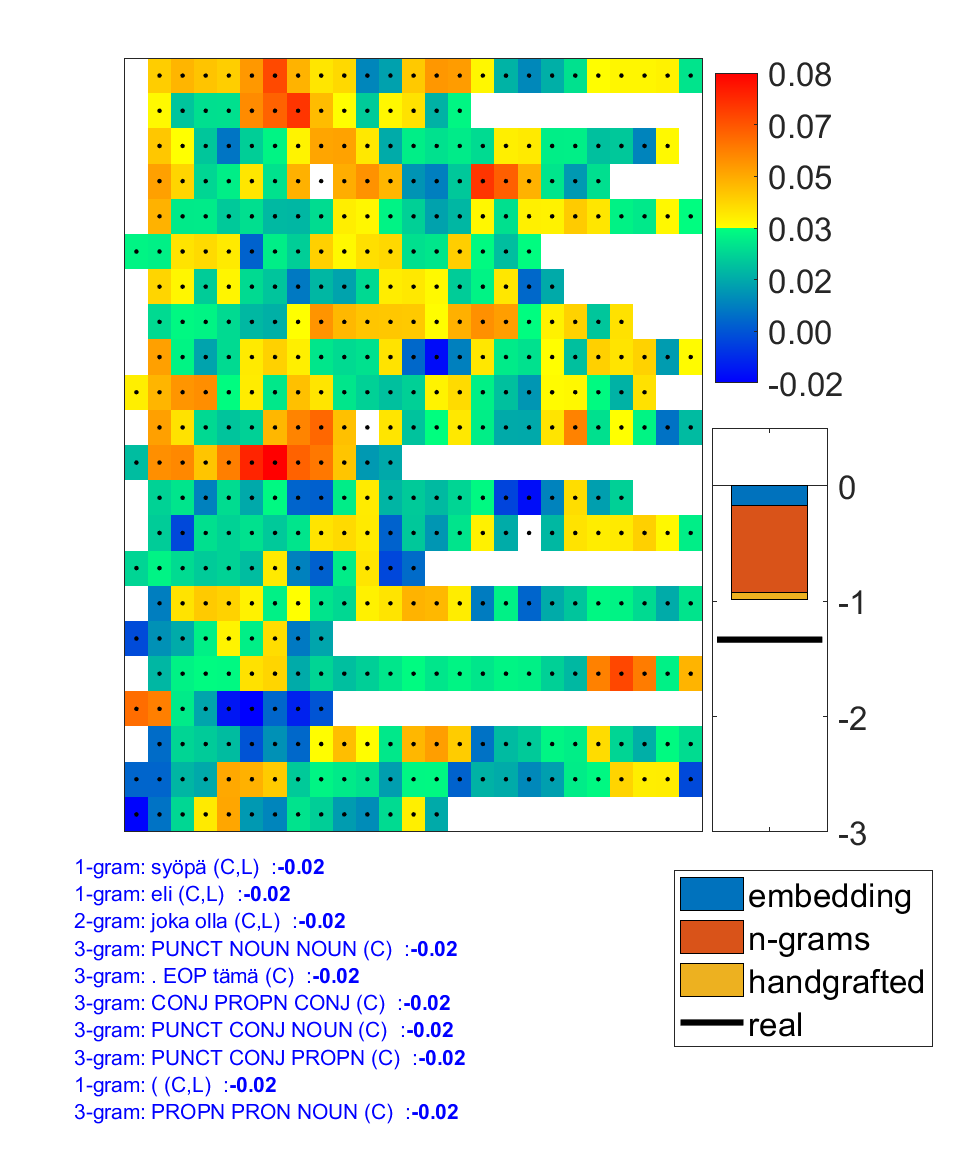  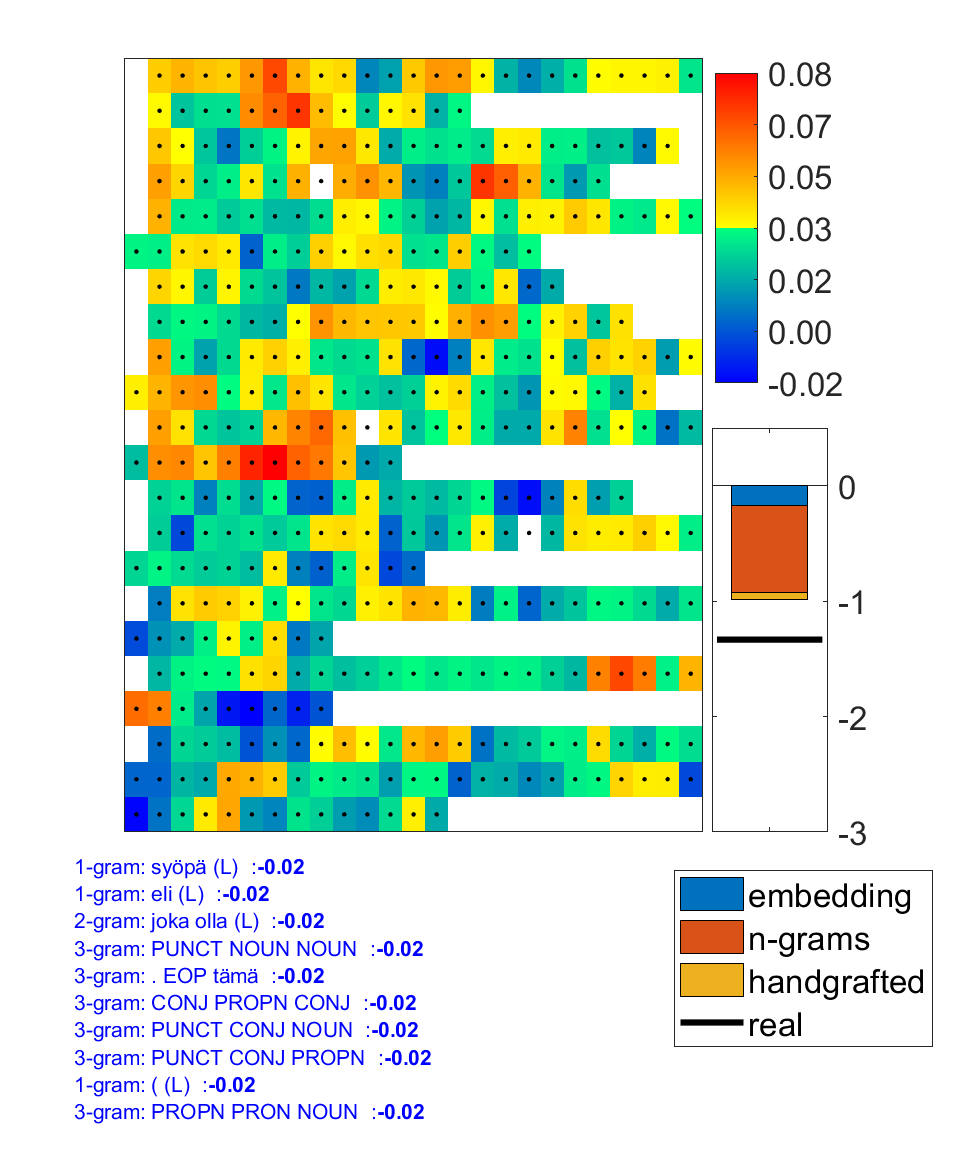 |

**Figure S9.** Similar to Fig. S8, but for a text sample with generally *low ratings* (1142 tokens).

# References

1. Zupanc K, Bosnić Z. Advances in the field of automated essay evaluation. Inform. 2015;39: 383–395.

2. Hardalov M, Koychev I, Nakov P. In Search of Credible News. In: Dicheva D, Dochev D, editors. ICNLSP 2018: 2nd International Conference on Natural Language and Speech Processing. Berlin, Heidelberg: Springer Berlin Heidelberg; 2016. doi:10.1007/978-3-642-15431-7

3. Hassan N, Arslan F, Li C, Tremayne M. Toward Automated Fact-Checking: Detecting check-worthy factual claims by ClaimBuster. 23rd ACM SIGKDD International Conference on Knowledge Discovery and Data Mining. New York, New York, USA: ACM Press; 2017. pp. 1803–1812. doi:10.1145/3097983.3098131

4. Kiritchenko S, Zhu X, Mohammad SM. Sentiment analysis of short informal texts. J Artif Intell Res. 2014;50: 723–762. doi:10.1613/jair.4272

5. Laippala V, Kanerva J, Missil A. Towards the Classification of the Finnish Internet Parsebank: Detecting Translations and Informality. Nodalida. 2015. pp. 107–116.

6. Lilleberg J, Zhu Y, Zhang Y. Support vector machines and Word2vec for text classification with semantic features. 2015 IEEE 14th International Conference on Cognitive Informatics & Cognitive Computing (ICCI*CC). 2015. pp. 136–140. doi:10.1109/ICCI-CC.2015.7259377

7. Ott M, Choi Y, Cardie C, Hancock JT. Finding Deceptive Opinion Spam by Any Stretch of the Imagination. Proceedings of the 49th Annual Meeting of the Association for Computational Linguistics. New York, New York, USA: ACM Press; 2011. pp. 309–319. doi:10.1145/2567948.2577293

8. Quijano-Sánchez L, Liberatore F, Camacho-Collados J, Camacho-Collados M. Applying automatic text-based detection of deceptive language to police reports: Extracting behavioral patterns from a multi-step classification model to understand how we lie to the police. Knowledge-Based Syst. 2018;149: 155–168. doi:10.1016/j.knosys.2018.03.010

9. Yu H, Hatzivassiloglou V. Towards answering opinion questions. Proceedings of the 2003 conference on Empirical methods in natural language processing -. Morristown, NJ, USA: Association for Computational Linguistics; 2003. pp. 129–136. doi:10.3115/1119355.1119372

10. Zesch T, Wojatzki M, Scholten-Akoun D. Task-Independent Features for Automated Essay Grading. Proc Build Educ Appl Work NAACL. 2015; 224–232. doi:10.3115/v1/W15-0626

11. Zhang D, Xu H, Su Z, Xu Y. Chinese comments sentiment classification based on word2vec and SVMperf. Expert Syst Appl. 2015;42: 1857–1863. doi:10.1016/j.eswa.2014.09.011

12. Manning CD, Raghavan P, Schütze H. An introduction to information retrieval. An introduction to information retrieval. Cambridge University Press; 2009.

13. Wang S, Manning C. Baselines and Bigrams: Simple, Good Sentiment and Topic Classification. Proc 50th Annu Meet Assoc Comput Linguist. 2012; 90–94.

14. Tantug AC. Document Categorization with Modified Statistical Language Models for Agglutinative Languages. Int J Comput Intell Syst. 2010;3: 632–645. doi:10.1080/18756891.2010.9727729

15. Mikolov T, Corrado G, Chen K, Dean J. Efficient Estimation of Word Representations in Vector Space. Proc Int Conf Learn Represent (ICLR 2013). 2013; 1–12. doi:10.1162/153244303322533223

16. De Boom C, Van Canneyt S, Demeester T, Dhoedt B. Representation learning for very short texts using weighted word embedding aggregation. Pattern Recognit Lett. 2016;80: 150–156. doi:10.1016/j.patrec.2016.06.012

17. Lai S, Xu L, Liu K, Zhao J. Recurrent Convolutional Neural Networks for Text Classification. Twenty-Ninth AAAI Conf Artif Intell. 2015; 2267–2273.

18. Kim HK, Kim H, Cho S. Bag-of-concepts: Comprehending document representation through clustering words in distributed representation. Neurocomputing. 2017;266: 336–352. doi:10.1016/j.neucom.2017.05.046

19. Xing C, Wang D, Zhang X, Liu C. Document classification with distributions of word vectors. 2014 Asia-Pacific Signal and Information Processing Association Annual Summit and Conference, APSIPA 2014. 2014. doi:10.1109/APSIPA.2014.7041633

20. Yang D, Lavie A, Dyer C, Hovy E. Humor Recognition and Humor Anchor Extraction. Empirical Methods in Natural Language Processing. 2015. pp. 2367–2376.

21. Cozma M, Butnaru AM, Ionescu RT. Automated essay scoring with string kernels and word embeddings. Proceedings of the 56th Annual Meeting of the Association for Computational Linguistics. 2018. pp. 503–509.

22. Joulin A, Grave E, Bojanowski P, Mikolov T. Bag of Tricks for Efficient Text Classification. 2016; doi:1511.09249v1

23. Li Y, Zhang L, Ma Y, Singh DJ. Character-level Convolutional Networks for Text Classification. Advances in Neural Information Processing Systems. 2015.

24. Tang D, Qin B, Liu T. Document Modeling with Gated Recurrent Neural Network for Sentiment Classification. Proceedings of the 2015 Conference on Empirical Methods in Natural Language Processing. 2015. pp. 1422–1432. doi:10.18653/v1/D15-1167

25. Kaiser E, Trueswell JC. Putting Things in Context: Sentence Processing in Languages with Flexible Word Order. Univ Pennsylvania Work Pap Linguist. 2003;9: 105–121.

26. Korenius T, Laurikkala J, Järvelin K, Juhola M. Stemming and lemmatization in the clustering of finnish text documents. Proceedings of the Thirteenth ACM conference on Information and knowledge management - CIKM ’04. New York, New York, USA: ACM Press; 2004. p. 625. doi:10.1145/1031171.1031285

27. Bojanowski P, Grave E, Joulin A, Mikolov T. Enriching Word Vectors with Subword Information. Transactions of the Association for Computational Linguistics. 2017. p. 135+146. doi:10.1017/S0140525X16001837

28. Pisarevskaya D, Litvinova T, Litvinova O. Deception Detection for the Russian Language: Lexical and Syntactic Parameters. Natural Language Processing and Information Retrieval Workshop. 2017. pp. 1–10.

29. Carvalho E, Amorim F De, Veloso A. A Multi-aspect Analysis of Automatic Essay Scoring for Brazilian Portuguese. Proceedings of the Student Research Workshop at the 15th Conference of the European Chapter of the Association for Computational Linguistics. 2017. pp. 94–102.

30. Kakkonen T, Myller N, Sutinen E. Applying Part-of-Seech Enhanced LSA to Automatic Essay Grading. Science (80- ). 2006; 500–504.

31. Kakkonen T, Sutinen E. Automatic Assessment of the Content of Essays based on Course Materials. Inf Technol Res Educ. 2004; 500–504. doi:10.1109/ITRE.2004.1393660

32. Zhou Z-H. Ensemble Methods: Foundations and Algorithms [Internet]. Chapman and Hall; 2012.

33. Attali Y, Burstein J. Automated Essay Scoring With e-rater® V.2. J Technol Learn Assess. 2006;4. doi:10.1136/bjsports-2017-098359

34. Napoles C, Callison-Burch C. Automatically Scoring Freshman Writing: A Preliminary Investigation. Proceedings of the Tenth Workshop on Innovative Use of NLP for Building Educational Applications. Stroudsburg, PA, USA: Association for Computational Linguistics; 2015. pp. 254–263. doi:10.3115/v1/W15-0629

35. Snyder B, Barzilay R. Multiple aspect ranking using the good grief algorithm. Proc NAACL HLT. 2007; 300–307. doi:10.1.1.129.4132

36. Smon H. Neural networks: a comprehensive foundation. Tsinghua Univ Press Beijing Song F, Gao X, Liu S Dimens Reduct Stat pattern Recognit low loss Dimens reduction Chin J Comput. 2001;28: 19151922Sun.

37. Chen T, Guestrin C. XGBoost. Proceedings of the 22nd ACM SIGKDD International Conference on Knowledge Discovery and Data Mining - KDD ’16. New York, New York, USA: ACM Press; 2016. pp. 785–794. doi:10.1145/2939672.2939785

38. Hastie T, Tibshirani R, Friedman J. The elements of statistical learning [Internet]. 2nd ed. Elements. Springer; 2009. doi:10.1007/b94608

39. Li S, Huang C-R, Zhou G, Lee SYM. Employing personal/impersonal views in supervised and semi-supervised sentiment classification. ACL ’10 Proc 48th Annu Meet Assoc Comput Linguist. 2010; 414–423.

1. <https://github.com/tmikolov/word2vec> [↑](#footnote-ref-1)
2. <https://github.com/dmlc/xgboost> [↑](#footnote-ref-2)
3. The mean correlation of coefficient vectors between the full data model and median over 10-fold models was 0.988, hence this had no effect on key results and conclusions [↑](#footnote-ref-3)
